# *Lavandula angustifolia* mill. (Lamiaceae) ethanol extract and its main constituents as promising agents for the treatment of metabolic disorders: chemical profile, *in vitro* biological studies, and molecular docking

**DOI:** 10.1080/14756366.2023.2269481

**Published:** 2023-10-18

**Authors:** Rosa Tundis, Fedora Grande, Maria A. Occhiuzzi, Vincenzo Sicari, Monica R. Loizzo, Anna R. Cappello

**Affiliations:** a Department of Pharmacy, Health and Nutritional Sciences, University of Calabria, Rende, Italy; b Department of Agraria, Mediterranean University of Reggio Calabria, Reggio Calabria, Italy

**Keywords:** Lavender, UHPLC-DAD analysis, enzyme inhibition, *in silico* study, antioxidant activity

## Abstract

*Lavandula angustifolia* Mill. (lavender) is one of the most used medicinal plants. Herein, we chemically characterised and investigated the antioxidant properties and the capability to inhibit key enzymes for the treatment of type 2 diabetes (TD2) and obesity such as pancreatic lipase, α-glucosidase, and α-amylase of the ethanolic extract of two lavender samples (La1 and La2) from southern Italy. Both extracts significantly inhibited α-glucosidase, while La1 inhibited α-amylase and lipase more effectively than La2. To investigate whether these properties could be due to a direct interaction of the main constituents of the extracts with the targeted enzymes, molecular docking studies have been performed. As a result, the selected compounds were able to interact with the key residues of the binding site of the three proteins, thus supporting biological data. Current findings indicate the new potential of lavender ethanolic extract for the development of novel agents for T2D and obesity.

## Graphical Abstract

**Figure:**
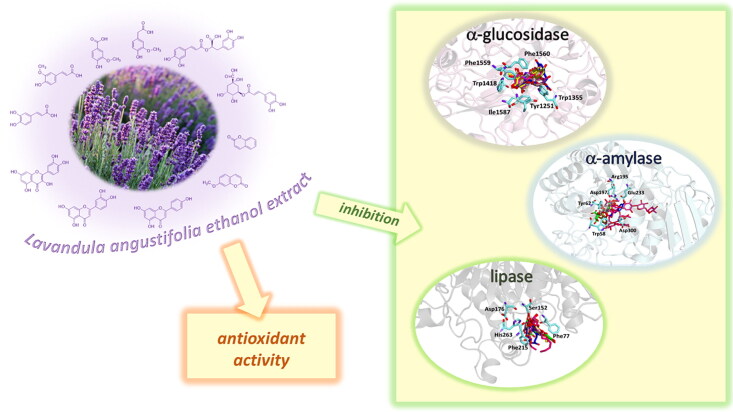

## Introduction

Noncommunicable diseases (NCDs), including diabetes mellitus, stroke, cancer, heart disease, and chronic respiratory disorders, are the leading cause of mortality worldwide. Common and variable risks underlie the major NCDs. They mainly include unhealthy diet, raised blood sugar, tobacco, harmful use of alcohol, low physical activity, overweight/obesity, and increased values of blood pressure and cholesterol.

Diabetes mellitus is a metabolic disease widely spread and rapidly increasing in the world, characterised by chronic hyperglycaemia, which results from defects in insulin secretion and/or insulin action^1^^,^^2^. Type 2 diabetes (T2D) is the principal form of diabetes, as an estimated about 90% of patients are diagnosed with this form. Its incidence continues to increase, and it is expected by 2035 that there will be more than 590 million patients with this disorder^1^^,^^3^. Most of the remaining patients are affected by type 1 diabetes (T1D), although other rare types of diabetes exist. T2D is essentially caused by impaired production and secretion of insulin by pancreatic beta cells, as well as by peripheral tissue insulin resistance.

T2D and obesity have an inter-dependent relationship, in fact, at the T2D diagnosis, approximately 90% of patients are obese or overweight. In recent years, the number of individuals affected by T2D has more than doubled, and the increased in the global burden of T2D is thought to be largely due to an increase in obesity, another complex disease of great public health significance worldwide. Additionally, weight loss is associated with a better prognosis for overweight or obeseT2D patients. A more beneficial glycaemic control has been documented in T2D patients who have lost weight, whereas excess body weight is associated with an increased risk of cardiometabolic complications, which are the leading causes of morbidity and mortality in T2D and obese individuals.

Due to the high prevalence of T2D and obesity, various therapeutic approaches have been attempted for their management, including the inhibition of key enzymes such as α-glucosidase and α-amylase and pancreatic lipase.

Starch is absorbed by the hydrolytic action of α-amylase followed by the action of intestinal α-glucosidase enzyme. Consequently, α-amylase inhibitors by modulating blood glucose levels after a meal may be considered effective chemotherapeutic tools for the treatment of diabetes. α-Glucosidase is a carbohydrate-hydrolysing enzyme secreted from the intestinal chorionic epithelium. The inhibition of this enzyme can promote a delay in carbohydrate digestion so preventing an excessive glucose absorption^4^. Currently, α-glucosidase inhibitors, such as acarbose, nojirimycin, etc. are successfully used to control the glucose level of diabetic patients.

Pancreatic lipase is the enzyme responsible for the absorption of dietary fats through the breakdown of triacylglycerols^5^. Inhibitors of this enzyme have recently attracted research interest due to their anti-obesity activity by delaying the lipolytic process. This action would lead to a decrease in lipid absorption and thus protect the pancreas, which will restore regular insulin production from the β cells^4^^,^^5^. Currently, the United States Food and Drug Administration has approved four drugs that act as pancreatic lipase inhibitors useful for weight control by promoting increased energy consumption and appetite suppression^6^. However, existing drugs are endowed with several undesired effects, and, for this reason, the development of alternative new agents to treat TD2 and obesity with fewer side effects and higher efficacy is still demanding. According to recent reports, many plant extracts, such as *Morus alba*, *Garcinia cambogia*, and *Plantago psyllium*, can modulate fat and carbohydrate metabolism regulating body weight^7^. These herbal medicines obtained great interest due to their effectiveness and minimal side effects.

In our ongoing efforts to evaluate the potentiality of the ethanolic extracts obtained by exhaustive maceration of *Lavandula angustifolia* Miller (lavender) to treat type 2 diabetes and obesity^8^, the present study was conducted to investigate the antioxidant and the inhibitory activity of key enzymes such as α-glucosidase, α-amylase, and pancreatic lipase together with the analysis of the chemical profile. In particular, the activity of the extracts in regulating the different mechanisms involved in the key features of T2D and obesity was investigated. Furthermore, molecular docking studies were conducted to establish whether these properties could be correlated with a direct interaction between the compounds contained in the extracts and the three enzymes.

Two samples of *L. angustifolia* have been collected in Pollino’s National Park. For the presence of some endemic species and rare plant species composition, this is a distinctive area in the Mediterranean basin. In fact, this national park covers an area of 192,500 ha across Basilicata and Calabria regions (Southern Italy) and is mainly characterised by a mountainous morphology, with three main massifs namely Pollino massif, located in the heart of the Park, Orsomarso mountain range in the south-west, and Monte Alpi in the north. The vegetation’s distinguishing feature is the great variety of species bearing witness to the variety and vastness of the territory and the different climatic conditions influencing it.

*L. angustifolia* is one of the most important species in the *Lavandula* genus^9^. Its natural distribution ranges from France, Italy, and Spain where it occurs as a montane species generally over 1500 m. It is cultivated also in Bulgaria, Albania, Hungary, Montenegro, the United Kingdom, the Netherlands, Russia, China, the United States, Argentina, and Australia.

In European traditional medicine, lavender preparations are used for their spasmolytic, carminative, stomachic, and diuretic effects. The lavender essential oil has been used for the treatment of candida infections, asthma, laryngitis, and sinusitis but also as a flavour component in food products, including aromatic vinegars, beverages, baked goods, candy, gelatines, frozen dairy desserts, and puddings^10^^,^^11^. Moreover, fresh flowers are added to vinegar, herbal teas, jams, and ice cream^12^.

*L. angustifolia* has been extensively studied for its chemical composition and biological properties. Most of these studies focused on its essential oil that has reported to possess sedative, analgesic, local anaesthetic, anticonvulsant, and anti-inflammatory effects^13–17^.

Some studies described the extraction of the polar constituents of *L. angustifolia*. As previously reported^18^, many of these studies used traditional methods such as maceration, digestion or infusion with alcohol hydro-alcoholic mixtures or water as solvent. However, among them the best results in terms of extraction yield were obtained by using maceration.

To the best of our knowledge, this is the first study dealing with the *in vitro* inhibitory effects of lavender ethanolic extract on these enzymes. In fact, only two studies analysed the potentiality of *L. angustifolia* in the treatment of TD2 and obesity. The essential oil of lavender has been previously investigated for its α-glucosidase inhibitory activity^19^. In another work, the methanol (80%) extract of lavender from Jordan exhibited a pancreatic lipase inhibitory activity^20^.

## Materials and methods

### Chemicals and reagents

Solvents of analytical grade were obtained from VWR International s.r.l. (Milan, Italy) while solvents used for Ultra-high performance liquid chromatography (UHPLC)-diode array detector (DAD) were purchased from Carlo Erba s.r.l. (Milan, Italy). Acarbose from *Actinoplanes* sp. was purchased from Serva (Heidelberg, Germany). α-Glucosidase from *Saccharomyces cerevisiae* (EC 3.2.1.20), α-amylase from porcine pancreas (EC 3.2.1.1), pancreatic lipase, apigenin, caffeic acid, chlorogenic acid, coumarin, herniarin, ferulic acid, luteolin, homovanillic acid, *p*-coumaric acid, quercetin, rosmarinic acid, and vanillinic acid, ascorbic acid, propyl gallate, butylated hydroxytoluene (BHT), quercetin, Tween 20, ascorbic acid, sodium potassium tartrate, sodium chloride, sodium carbonate, Folin-Ciocalteu reagent, 2,2-diphenyl-1-picrylhydrazyl (DPPH), 2,4,6-tripyridyl-*s*-triazine (TPTZ), 2,2′-azino-bis(3-ethylbenzothiazoline-6-sulfonic acid) diammonium salt, (ABTS) solution, sodium acetate, β-carotene, linoleic acid, peroxidase-glucose oxidase (PGO), sodium phosphate, maltose, 3,5-dinitrosalicylic acid, potato starch, and *o*-dianisidine colour reagent (DIAN) were purchased from Sigma-Aldrich s.r.l. (Milan, Italy).

### Plant materials and extraction procedure

The aerial parts of *Lavandula angustifolia* have been collected in June 2021 in Calabria (Southern Italy) in the area of Pollino National Park (WGS84: 39° 88′ 09″ N, 16° 08′ 39″ E) (sample La1) and in locality Campotenese (WGS84: 39° 87′ 04″ N, 16° 06′ 48″ E) (sample La2). Both these areas are at about 1000 m above sea level. Samples were collected by dr. Antonio Grisolia (Azienda agricola Grisolia, Italy) and identified by dr. Nicodemo G. Passalacqua, University of Calabria (Italy).

Both samples were examined to verify their integrity and the absence of any contamination and subjected to an extraction procedure. In particular, the fresh aerial parts of *L. angustifolia* (431.4 g and 488.3 g for La1 and La2, respectively) were extracted through maceration (3 L) with ethanol (3 × 48 h) at room temperature. Extractive solutions obtained from each sample were combined, filtered, and evaporated under reduced pressure in order to obtain the dry extracts that were stored in hermetically sealed brown glass bottles, and kept at 4 °C before chemical and biological analyses.

#### Total Phenols content (TPC)

The Total Phenols Content (TPC) was determined by using the Folin-Ciocalteu method in which lavender extracts (at the concentration of 1.5 mg/mL) were mixed with water, sodium carbonate 15% (w/v), and Folin-Ciocalteu reagent (a mixture of phosphomolybdate and phosphotungstate)^20^. After an incubation of 2 h at room temperature, the absorbance was read at 765 nm by using a UV-vis Jenway 6003 spectrophotometer (Milan, Italy). TPC was determined in triplicate and was reported as mg of chlorogenic acid (CA) equivalents/g of extract.

#### Total flavonoids content (TFC)

For the Total Flavonoids Content (TFC) determination, lavender extracts (at the concentration of 1.5 mg/mL) were added to distilled water and sodium nitrite 5% (w/v). After 5 min, aluminium chloride 10% (w/v) was added. After other 6 min, sodium hydroxide 1 M and water were added. Then, the absorbance was read at 510 nm. TFC was determined in triplicate and was reported as mg of quercetin equivalents (QE)/g of extract^21^.

### Ultra-high performance liquid chromatography (UHPLC) - diode array detector (DAD) analyses

The ultra-high performance liquid chromatography (UHPLC) coupled with diode array detection (DAD) analyses were performed by a Knauer instrument system (Asi Advanced Scientific Instruments, Berlin, Germany) equipped with PDA-1 (Photo Diode Array Detector) PLATIN blue (Knauer, Germany), a software Clarity 6.2 (Chromatography Station for MS Windows) and by using a Kinetex 2.6 µm Biphenyl 100 AÅ^22^.

The mobile phases consisted of (A) water/formic acid, 99.9:0.1, v/v) and (B) acetonitrile. Analyses were performed at a flow rate of 0.4 ml/min in gradients as follows: 0–3 min, 95% A and 5% B; 3–15 min, 95–60% A and 5–40% B; 15–15.5 min, 60–0% A and 40–100% B. Authentic standards were used to confirm the presence of apigenin, caffeic acid, chlorogenic acid, coumarin, herniarin, ferulic acid, luteolin, homovanillic acid, *p*-coumaric acid, quercetin, rosmarinic acid, and vanillic acid. The method was validated for a limit of quantification (LOQ) and limit of detection (LOD) defined as the lowest concentration in the standard solution with the percentage of the relative standard deviation (% RSD) ≤ 10% and calculated following the equations: LOD = SD × 3.3 and LOQ = SD × 10 (Table S1, Supplementary Materials).

### *In vitro* antioxidant assays

The activity of an antioxidant agent depends not only on its structural characteristics, but also on other factors, such as the physical state of the system, concentration, temperature, and substrate, as well as on the numerous micro-components that can act as pro-oxidants or in synergy. Furthermore, not a single *in vitro* antioxidant assay can reflect the real antioxidant capacity *in vivo*^23^. For this reason, it is desirable to test *in vitro* the antioxidant activity by using different methods. In the present work, the antioxidant activity of lavender extracts was evaluated by using the β-carotene bleaching test, and ABTS (2,2′-azino-bis(3-ethylbenzothiazoline-6-sulphonic acid) test, DPPH (2,2-diphenil-1-picrylhydrazyl) test, and ferric reducing antioxidant power (FRAP) as previously described^24^.

In the β-carotene bleaching assay, heat-induced oxidation of an emulsion system of β-carotene and linoleic acid was used. Linoleic acid forms a peroxyl radical that reacts with β-carotene to form a stable β-carotene radical. Then, the amount of β-carotene reduces in a testing solution. If an antioxidant is present in a testing solution, it reacts competitively with the peroxyl radical. Therefore, antioxidant effects are monitored by bleaching the colour at 470 nm. In brief, a mixture of linoleic acid (0.02 ml), β-carotene (0.2 mg/mL in chloroform), and Tween 20 (0.2 ml) was prepared. After evaporation of chloroform and dilution with water, 5 ml of the emulsion was transferred into different test tubes containing 0.2 ml of lavender extract at different concentrations in the range 50–1000 mg/mL. Tubes were then shaken and placed in a water bath at 45 °C for 60 min. The absorbance was measured at 470 nm using a UV-Vis Jenway 6003 spectrophotometer against a blank. The measurement was carried out immediately after their preparation (*t* = 0) and successively at *t* = 30 min and *t* = 60 min. The antioxidant activity (AA), measured in terms of successful bleaching β-carotene, was calculated by using the following equation: AA= [1 − (A_0_ − A_t_)/(A°_0_ − A°_t_)] × 100, where A_0_ and A°_0_ are the absorbance values at the initial incubation time for samples/standard and control, respectively, while A_t_ and A°_t_ are the absorbance values measured for samples/standard and control, respectively, at *t* = 30 min and *t* = 60 min. Propyl gallate was used as a positive control.

The radical scavenging activity was investigated by using two assays, namely ABTS and DPPH tests. Ascorbic acid was used as a positive control in both ABTS and DPPH assays.

In the ABTS test, a stable ABTS radical cation (which has a blue-green chromophore absorption) solution was produced (and left before the use for 12 h in the dark) by oxidation of ABTS with potassium persulfate prior to the addition of antioxidants. The antioxidant activity of lavender extracts is determined by the decolourization of the ABTS, by measuring the reduction of the radical cation as the percentage inhibition of absorbance at 734 nm.

The DPPH free radical is a stable organic nitrogen radical with a purple colour. When a DPPH solution is mixed with an antioxidant, its colour turns from purple to yellow of the corresponding hydrazine. The reducing ability of an antioxidant towards DPPH can be evaluated by monitoring the decrease of its absorbance at 527 nm. In brief, in this assay a mixture of DPPH solution (1.0 × 10^−4^ M) and lavender extracts at different concentrations was prepared and kept for 30 min in the dark.

The activity (A) was calculated by using the following equation: A = [(A_0_ − A_1_)/A_0_] × 100, where

A_0_ is the absorbance of the control (blank, without lavender extract) and A_1_ is the absorbance in the presence of the lavender extract.

In the FRAP assay, based on the reaction that involves 2,4,6-tripyridyl-s-triazine)-Fe^3+^ (TPTZ) complex, a mixture of TPTZ solution 10 mM (2.5 ml) in HCl 40 mM, FeCl_3_ 20 mM (2.5 ml) and acetate buffer 0.3 M (pH 3.6, 25 ml) was prepared. When a Fe^3+^-TPTZ complex is reduced to the Fe^2+^ form by an antioxidant under acidic conditions, a blue colour with an absorption maximum develops at 595 nm. Therefore, the antioxidant effect can be evaluated by monitoring the formation of a Fe^2+^-TPTZ complex with a spectrophotometer. The absorbance was read at 595 nm and butylated hydroxytoluene (BHT) was used as a positive control.

### α-Glucosidase inhibitory activity

To carry out the α-glucosidase inhibitory activity test, we prepared (1) the enzyme solution by adding α-glucosidase from *S. cerevisiae* (EC 3.2.1.20) (10 units/mg) in of ice-cold water (10 ml), (2) the maltose solution by dissolving 12 g of maltose in 300 ml of 50 mM sodium acetate buffer, (3) the *o*-dianisidine (DIAN) solution by dissolving 1 tablet in 25 ml of water, and (4) the peroxidase/glucose oxidase (PGO) system-colour reagent solution by dissolving 1 capsule in 100 ml of ice-cold water^21^.

In the first step, a mixture of lavender extract (at concentrations in the range 25–1000 µg/mL), maltose solution, and enzyme was incubated for 30 min at 37 °C. Then, perchloric acid solution (4.2% w/v) was added to stop the reaction. Successively, the generation of glucose was quantified by the reduction of DIAN. The supernatant of tubes of the first step (obtained after centrifugation) was mixed with PGO and DIAN solutions, and incubated for 30 min at 37 °C. Then, the absorbance was read at 500 nm. Acarbose, a drug commonly used to treat type 2 diabetes, was employed as positive control.

###  α-Amylase inhibitory activity

The potential inhibitory properties of α‐amylase by lavender extracts were explored as previously described^21^. In the α-amylase inhibitory activity test, lavender extracts were dissolved in ethanol at concentrations ranged from 25 to 1000 µg/mL. We prepared the enzyme solution by adding 1 mg of α-amylase from porcine pancreas (EC 3.2.1.1) in 10 ml of cold water, and the starch solution by stirring potato starch (0.125 g) in 25 ml of sodium phosphate buffer 20 mM and sodium chloride 6.7 mM at 65 °C for 15 min. The colorimetric reagent was prepared mixing a sodium potassium tartrate solution and 96 mM 3,5-dinitrosalicylic acid solution. Both control and extracts were added to the starch solution and left to react with the enzyme solution at 25 °C. The reaction was measured over 3 min. The generation of maltose was quantified by the reduction of 3,5-dinitrosalicylic acid to 3-amino-5-nitrosalicylic acid. This reaction that corresponds to a colour change from orange-yellow to red is detectable at 540 nm. In the presence of an α-amylase inhibitor, less maltose would be produced and the absorbance value would be decreased. Acarbose was used as a positive control.

### Pancreatic lipase inhibitory activity

To investigate the potential pancreatic lipase inhibitory activity of lavender extracts, a 96-well plate method was adopted^25^. Concisely, 4-nitrophenyl octanoate (NPC) (5 mM) in dimethyl sulfoxide solution and an aqueous solution of pancreatic lipase (1 mg/mL) and Tris-HCl buffer (pH 8.5) were prepared. Lavender extract at different concentrations ranged from 2.5 to 40 mg/mL was added to a well together with NPC, enzyme, and Tris‐HCl buffer, and the mixture was incubated for 30 min at 37 °C. Then, the absorbance was measured at 405 nm. Orlistat was used as a positive control.

### Molecular docking

Molecular docking was performed on the crystallographic structure of α-glucosidase, α-amylase and pancreatic lipase, corresponding to the entries 3TOP^26^ 4W93^27^ and 1LPB^28^ in the Protein Data Bank (PDB), respectively^29^^,^^30^.

Molecular structures of the twelve main ligands identified in the *L. angustifolia* extracts were built by using the modelling software Avogadro^31^. Docking calculations were performed by using AutoDock Vina 1.1.2^32^. Preliminary conversion of the structures from the PDB format was carried out by the graphical interface AutoDock Tools 1.5.6^33^. During the conversion, polar hydrogens were added to the crystallographic enzyme structures, whereas apolar hydrogens of the ligands were merged to the carbon atom they are attached to^34^. Full flexibility was guaranteed for the ligands, resulting in a number ranging from 0 to 11.

All compounds with an acidic nature were tested in the anionic form. The binding modes of the ligands were analysed through visual inspection, and intermolecular interactions were evaluated by using the automated protein-ligand interaction profiler, PLIP^35^ and MOE 20018.01 (Molecular Operating Environment)^36^.

### Statistical analysis

Experiments were performed in triplicate. Data are represented as the mean ± standard deviation.

Biological data were fitted through nonlinear regression in order to calculate the IC_50_ (concentration causing 50% inhibition) values. Graphs were built using GraphPad Prism version 4.0 for Windows software (GraphPad Software; San Diego, CA, USA). Data were analysed by One‐way analysis of variance (ANOVA) (SPSS 17.0 software, Chicago, IL, USA). Significant differences were calculated according to Tukey’s multiple range tests. Differences at **p < .01 were considered statistically significant.

## Results and discussion

### Extraction yield, soil physico-chemical parameters and chemical profile

Two samples of lavender (La1 and La2) were collected in Pollino’s National Park (southern Italy) and subjected to exhaustive maceration with ethanol. Extraction yields of 8.7 and 10.2% were obtained for La1 and La2, respectively. La1 extract exhibited the Total Phenolic Content (TPC) of the extracts of *L. angustifolia* was assessed and amounted to 27.9 mg/g and 26.8 mg/g for La1 and La2, respectively. The amounts were expressed as chlorogenic acid (CA) equivalents *per* g of extract. A Total Flavonoids Content (TFC) of 18.5 mg/g and 17.6 mg/g for La1 and La2, respectively. The amounts were expressed as quercetin (QE) equivalents/g of extract. Results evidenced as La1 exhibited the highest content in both TPC and TFC compared to La2. Previous studies have analysed the TPC and TFC of lavender polar extracts^37–39^. Dobros et al.^37^ extracted three cultivars of *L. angustifolia*, namely Elizabeth, Hidcote, and Betty’s Blue, by maceration with 50% ethanol, decoction, and Ultrasound-assisted extraction with 50% ethanol. TPC values ranged between 14.88 and 32.82 mg gallic acid equivalents (GAE)/g dried weight and TFC values in the range from 8.51 to 23.70 mg CA/g dried weight were found.

More recently, Caser et al.^39^ chemically characterised the fresh and dried lavender flower extracts obtained by ultrasound-assisted maceration and compared two drying procedures, such as hot-air drying (HA) and heat-pump drying (HP). TPC values in the range from 1268.43 mg GAE/100 g dry matter in fresh flowers to 595.35 mg GAE/100 g DM in HP flowers and 357.08 mg GAE/100 g dry matter in HA flowers were found.

Lavender extracts have been analysed by UHPLC-DAD. Data are reported in Table 1. Based on the literature data, we have selected seven phenolic acids, three flavonoids, and two coumarins as authentic markers. Both lavender extracts are characterised by a quite similar profile from a qualitative point of view. However, there are some interesting differences in the content of some constituents, particularly chlorogenic acid, homovanillic acid, rosmarinic acid, and quercetin.

**Table 1.** HPLC-DAD analysis of selected markers (mg/g extract) of lavender ethanolic extracts.

Compound	La1	La2
*Phenolic acids*		
Caffeic acid	2.5 ± 0.3	4.9 ± 0.3
Chlorogenic acid	75.1 ± 2.5	19.6 ± 1.4
Ferulic acid	0.3 ± 0.02	1.2 ± 0.1
Homovanillic acid	6.9 ± 0.6	13.5 ± 1.2
*p*-Coumaric acid	0.2 ± 0.02	2.2 ± 0.2
Rosmarinic acid	32.7 ± 2.1	17.2 ± 1.6
Vanillic acid	2.1 ± 0.1	3.6 ± 0.6
*Flavonoids*		
Apigenin	1.2 ± 0.3	3.4 ± 0.2
Luteolin	1.6 ± 0.1	2.2 ± 0.2
Quercetin	4.1 ± 0.5	Nd
*Coumarins*		
Coumarin	0.5 ± 0.2	1.4 ± 0.2
Herniarin	tr	tr

Data are reported as mean ± standard deviation (*n* = 3). nd: not detected. tr: trace (<0.1 mg/g).

Chlorogenic acid was found to be the most abundant compound in La1 with a content almost four times higher than that of La2, followed by rosmarinic acid with a content in La1 almost twice higher than that of sample La2. On the other hand, sample La2 exhibits a homovanillic acid content approximately twice higher than that found in La1. The flavonoid quercetin is present exclusively in the La1 sample with a content of 4.13 mg/g. Sample La2 is richer than La1 in caffeic acid, ferulic acid, *p*-coumaric acid, vanillic acid, apigenin and luteolin content.

In a previous job, phenolic acids and flavonoids were identified as the main classes of compounds in two cultivars of *L. angustifolia* namely “Blue River” and “Ellagance Purple” from Poland. In particular, phenolic acids, such as rosmarinic acid, ferulic acid and caffeic acid, and three flavonoids, namely apigenin, luteolin and quercetin, were identified^40^.

The extracts obtained by maceration with 50% ethanol, decoction, and ultrasound-assisted extraction with 50% ethanol of three cultivars of *L. angustifolia*, namely Elizabeth, Hidcote, and Betty’s Blue, analysed by HPLC, showed the presence as the most abundant constituents of rosmarinic acid (2.52–10.82 mg/g), ferulic acid glucoside (2.94–8.67 mg/g), caffeic acid (1.70–3.10 mg/g), morin (1.02–13.63 mg/g), coumarin (1.01–5.97 mg/g) and herniarin (1.05–8.02 mg/g)^37^.

Caser et al.^39^ chemically characterised the fresh and dried lavender flower ultrasound-assisted extracts identifying by HPLC seven compounds in fresh flower, namely caffeic acid, catechin, hyperoside, quercetin, dehydroascorbic acid, ellagic acid, and epicatechin, four constituents in the extract obtained after hot-air drying (epicatechin, ferulic acid, hyperoside, and quercitrin), and two in the extract obtained after heat-pump drying, such as caffeic acid and hyperoside.

The flavonoid quercetin (664.06 mg/100 g DM) together with some other compounds was detected only in fresh flowers. Analysing results from this work and comparing these data with our results we can conclude that fresh lavender flower polar extracts have an interesting phytochemical profile. Processing methods may be useful to prolong their utilisation without compromising their characteristics. However, hot-air drying and heat-pump drying showed to affect the content of bioactive compounds differently.

### Antioxidant activity

To assess the potential antioxidant activity of lavender samples, four tests were herein applied. Results are reported in Table 2. A remarkable radical scavenging activity in the ABTS test was found. In fact, both extracts possessed a greater activity (IC_50_ values of 0.5 and 0.4 µg/mL for La1 and La2, respectively) than the positive control ascorbic acid (IC_50_ value of 1.1 µg/mL).

**Table 2.** Antioxidant effects of lavender ethanolic extracts.

Antioxidant assay	La1	La2	Sign.
ABTS test (IC_50_ µg/mL)	0.5 ± 0.02	0.4 ± 0.03	
DPPH test (IC_50_ µg/mL)	3.9 ± 0.6^a^	4.4 ± 0.4^b^	**
FRAp^ test (µM Fe(II)/g)	50.7 ± 1.3^b^	31.4 ± 1.1^a^	**
β-Carotene bleaching test (IC_50_ µg/mL)			
- Incubation 30 min	12.1 ± 1.4^a^	29.6 ± 1.9^b^	**
- Incubation 60 min	9.1 ± 1.6^b^	6.8 ± 1.2^a^	**

Data are expressed as media ± SD (standard deviation) (*n* = 3). ABTS test: positive control ascorbic acid, IC_50_ di 1.1 ± 0.3 µg/mL; DPPH test: positive control ascorbic acid, IC_50_ of 5.2 ± 0.6 µg/mL; FRAP test: positive control BHT, 8.6 ± 0.7 µM Fe(II)/g, at the concentration of 0.3 mg/mL; β-Carotene bleaching test: positive control propyl gallate, IC_50_ di 1.3 ± 0.4 µg/mL after 30 min of incubation and IC_50_ di 1.1 ± 0.3 µg/mL after 60 min of incubation. Differences within and between groups were evaluated by one-way ANOVA followed by Tukey’s multiple range test. Results followed by different letters in a same column are significantly different at **p < 0.01. ^Samples tested at the concentration of 0.3 mg/mL.

A promising FRAP was also observed. Indeed, both lavender ethanolic extracts were more active than BHT. The FRAP test is the only assay that directly measures antioxidant agents in a sample compared to other tests that measure free radicals’ inhibition^41^. The values obtained in the FRAP test represent the corresponding concentration of electron-donating antioxidants with the reduction in the ferric iron to the ferrous ion. FRAP is deemed a suitable assessment for total antioxidants in plants consumed by humans because the only compounds with which FRAP does not react with are the thiols. Only a limited amount of glutathione in plants is absorbed by humans.

The highest inhibition of lipid peroxidation in the β-carotene bleaching test was recorded with La2 with an IC_50_ value of 6.8 µg/mL after 60 min of incubation.

Polyphenols are known to act as antioxidant agents not only because of their ability to donate electrons or hydrogen atoms but also because of their ability to prevent the oxidation of various food ingredients, particularly including oils and fatty acids^42^. Chlorogenic acid and rosmarinic acids mainly characterised lavender ethanolic extracts, together with some flavonoids such as quercetin, apigenin and luteoilin. Chlorogenic acid (5-*O*-caffeoylquinic acid) has antioxidant activity and can alleviate oxidative stress in various *in vitro* and *in vivo* models^43–47^. The protective effect of cholorogenic acid against oxidative stress has been investigated in the PC12 cell line with respect to lipid peroxidation, ROS formation, glutathione depletion, and prevention of cell death^46^. The phenolic acid showed to counteract lipid peroxidation and to eliminate ROS induced by all stressors. Chlorogenic acid showed protection against H_2_O_2_-induced oxidative stress also in human HaCaT cell line^43^. Moreover, it is effective at reducing the damage caused by ultraviolet light (UVB) exposure.

The photo-oxidation protection offered by chlorogenic acid in a mouse epidermal cell line and human HaCaT keratinocytes was related to the effects of the molecule to trigger the induction of phase II enzyme activities and Nrf2 transactivation^43^^,^^47^.

Several studies highlighted the great antioxidant activity of rosmarinic acid^48–51^. Rosmarinic acid exhibited free radicals scavenging activity, suppressed lipid peroxidation, increased the expression and activity of catalase, haem oxygenase-1, superoxide dismutase, and the Nrf2transcription, which are decreased by UVB radiation^52^. The antioxidant activity was also related to the presence of flavonoids. Tian et al.^53^ reported the DPPH radical scavenging ability of both quercetin and luteolin with IC_50_ values of 1.84 and 2.10 µg/mL, respectively. Values of 0.50, 0.59, and 0.82 µg/mL for quercetin, luteolin, and apigenin, respectively, were measured against ABTS^+^·radical whereas FRAP values in the range 0.01–0.04 mmol Fe^2+^/mg/mL for apigenin and luteolin, respectively was found. Moreover, luteolin, a natural and safe antioxidant phytochemical with less pro-oxidant potential than quercetin, showed a DPPH radical scavenging activity more potent than vitamin C (IC_50_ value of 3.03 µg/mL)^54^. The antioxidant capacity of flavonoids is closely related to the number of hydroxyl groups in their skeleton. Thus quercetin, a flavonoid with the highest number of hydroxyl groups among the compounds present in the lavender extract, which is characterised by five hydroxyl groups, with the same content, will contribute more to the antioxidant activity than flavonoids with a lower number of hydroxyl group, at least in radical scavenging essays e.g. DPPH and ABTS. Another important aspect to consider is the position of the hydroxyls in the skeleton since the group without steric hindrance can interact more easily with the radicals^42^.

### Effects of lavender ethanolic extracts on key enzymes involved in type 2 diabetes and obesity

Inhibitors of α-amylase and α-glucosidase can reduce post-prandial hyperglycaemia by delaying the digestion of carbohydrates and, for this reason, are valuable agents in the treatment of TD2. In the same way, the pancreatic lipase inhibition is useful for the treatment of obesity. Recently, more attention is paid by the scientific community to the study of the biological activities of plant derived extracts by applying enzyme inhibition tests for the discovery of potential anti-diabetic and anti-obesity drugs. In this context, herein, we have investigated the potential activity of La1 and La2 extracts against these enzymes. Both extracts inhibited the enzymes in a concentration-dependent manner. The IC_50_ values are reported in Table 3.

**Table 3.** Enzymes inhibitory activity (IC_50_, µg/mL) of lavender ethanolic extracts.

Assay	La1	La2	*Sign.*
α-Glucosidase	2.5 ± 0.2^a^	2.1 ± 0.3^a^	
α-Amylase	6.8 ± 0.6^a^	14.8 ± 2.4^b^	**
Pancreatic lipase	30.5 ± 3.1^a^	44.5 ± 3.2^b^	**

Positive controls: α-glucosidase inhibitory activity test, acarbose (IC_50_ 35.5 ± 1.2 µg/mL); α-amylase inhibitory activity test, acarbose (IC_50_ 50.2 ± 1.9 µg/mL); lipase inhibitory activity test, orlistat (IC_50_ 37.1 ± 1.1 µg/mL). Data are expressed as media ± standard deviation (*n* = 3). Differences within and between groups were evaluated by One-way ANOVA followed by Tukey’s multiple range test. Results followed by different letters in a same column are significantly different at **p < .01.

Interesting results were obtained against α-glucosidase with both lavender samples that exhibited IC_50_ values of 2.5 and 2.1 µg/mL for La1 and La2, respectively. La1 was more active than La2 in inhibiting α-amylase with IC_50_ values of 6.8 µg/mL and 14.8 µg/mL. Obtained data are better than that the positive control acarbose in both α-glucosidase and α-amylase inhibitory assays (IC_50_ of 35.5 and 50.2 µg/mL, respectively).

The prevalence of obesity is alarmingly increasing in the last few decades and leading to many serious public health concerns worldwide. The dysregulated lipid homeostasis due to various genetic, environmental and lifestyle factors is considered one of the critical putative pathways mediating obesity. Nonetheless, the scientific advancements unleashing the molecular dynamics of lipid metabolism have provided deeper insights on the emerging roles of lipid hydrolysing enzymes, including pancreatic lipase. It is hypothesised that inhibiting pancreatic lipase would prevent the breakdown of triglyceride and delays the absorption of fatty acids into the systemic circulation and adipocytes. Whilst orlistat is the only conventional pancreatic lipase enzyme inhibitor available in clinics, identifying safe clinical alternatives from plants to inhibit pancreatic lipase has been considered a significant advancement. Consequently, plants which have shown significant potential to combat obesity are now revisited for its abilities to inhibit pancreatic lipase.

In this regard, our data are of great interest. In fact, La1 inhibited lipase with an IC_50_ of 30.5 µg/mL, value better than the positive control orlistat.

Several studies have described the *in vitro* inhibition of α-amylase, α-glucosidase and lipase induced by phenolic acids and flavonoids^55–65^. The IC_50_ values of some identified constituents of *L. angustifolia* extracts are reported in Table 4.

**Table 4.** Enzymes inhibitory activity of the main identified phenolic acids and flavonoids of lavender extracts.

	α-Glucosidase		α-Amylase		Lipase	
Compound	IC_50_ (mM)	Ref	IC_50_ (mM)	Ref	IC_50_ (mM)	Ref
Caffeic acid	27.6 × 10^−3^	53	20.4 × 10^−3^	53	0.18	54
*p*-Coumaric acid	> 30	55	> 30	56	0.17	57
Chlorogenic acid	10.6 × 10^−3^	58	5.2 × 10^−3^	58	0.27	54
Ferulic acid	4.9	55	9.5	55	0.12	59
Rosmarinic acid	12.0 × 10^−3^	58	2.5 × 10^−3^	58	0.35	54
Vanillic acid	0.27	60	27.9	60	8.9	60
Apigenin	> 0.20	61	> 0.5	61	0.38	62
Luteolin	0.10	58	0.07	58	0.099	63
Quercetin	7 × 10^−3^	61	0.50	61	6.1 × 10^−3^	57

In particular, luteolin and quercetin resulted non-competitive α-amylase inhibitors. The formation of the enzyme-flavonoid complex could determine the alteration of the active site of the enzyme and inhibiting the enzyme-endogenous ligand interaction, and thus causing the inhibition of the enzymatic activity^66^. In the last decade numerous studies have reported the ability of flavonoids to inhibit α-glucosidase enzyme.

From the analysis of structure-activity relationships the decisive role of hydroxyls at C-3 and C-4′ as well as a double bond between position C-2 and C-3 merged. At the same time the substitution of the sugar in any position on the aglycone resulted in a reduction of the inhibitory activity. The importance of the hydroxyl groups is probably related to the ability of these groups to facilitate the electrostatic interaction between the phytochemical and the enzyme, making the flavonols inhibitors stronger than their flavonic analogues^67^. The study of structure-activity relationships between flavonoids and lipase enzyme evidenced the determinant role of the structure of the C ring. In fact, the reduction of the double bond between C2 and C3, and the absence of the C = O group in the C ring resulted in a strong reduction of the enzyme inhibitory activity. Likewise, the hydroxylation on ring A and B of flavones increased the lipase inhibitory activity, while glycosylation weakened^65^.

### Molecular docking

Nowadays, the application of computational techniques offers a number of advantages in rationalising the route towards the discovery of novel drugs or the identification of the biological properties of compounds, both of natural or synthetic origin, to be used in the treatment or the supplementation of several diseases^68^^,^^69^. In particular, molecular docking allows to fit a ligand into a binding site by combining and optimising variables like steric, hydrophobic and electrostatic complementarity.

Furthermore, one of the main goals of molecular docking is the identification of the most energetically favourable conformation of a ligand in the binding site of the target molecule (the more negative the binding energy, the better is the ligand-target stability).

**Figure 1.**
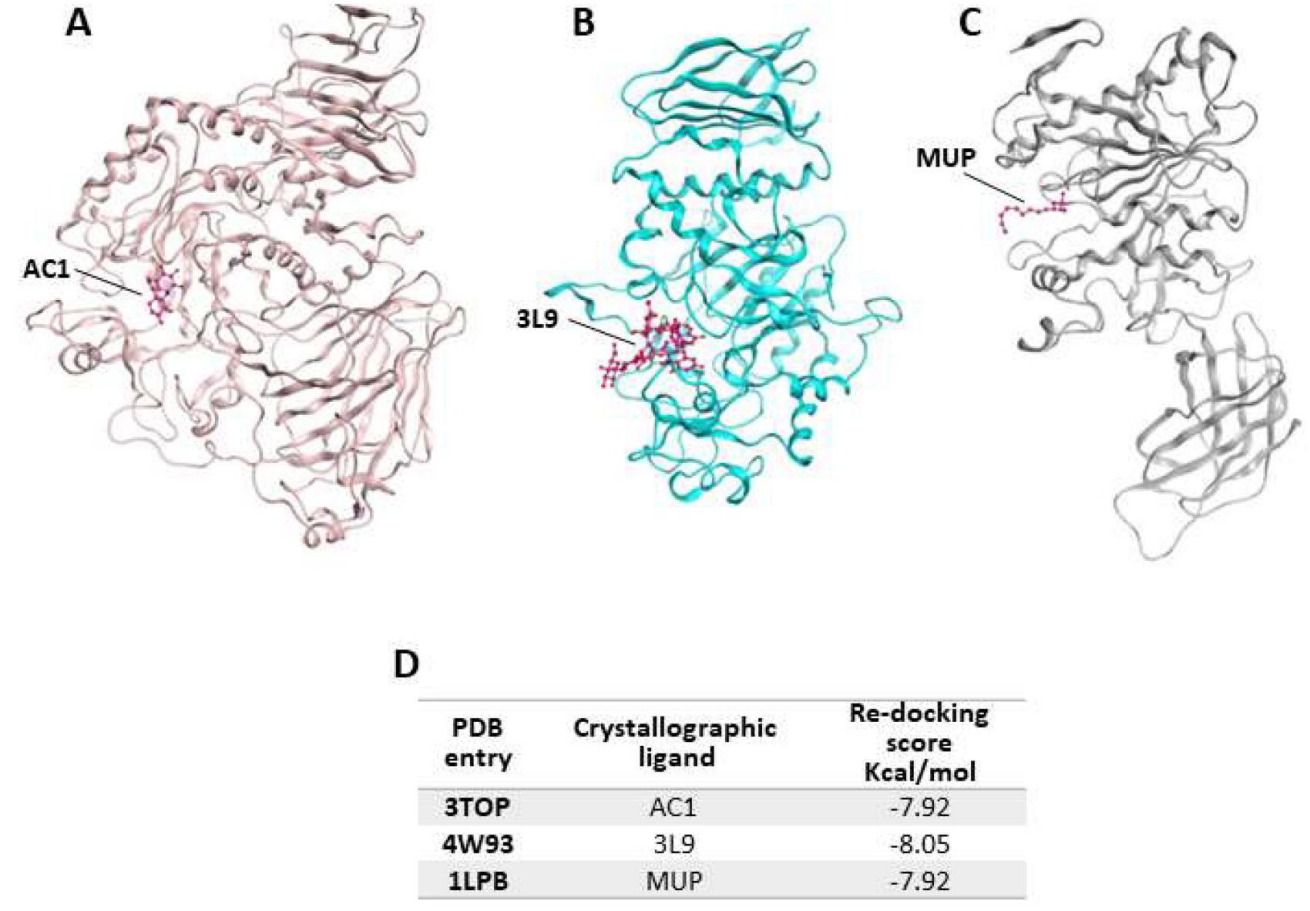
Crystallographic structure of α-glucosidase, α-amylase and pancreatic lipase, corresponding to the PDB entries (A) 3TOP, (B) 4W93 and (C) 1LPB; (D) re-docking score values for crystallographic ligands.

Accordingly, in order to verify whether the biological activity of the extract could be attributed to a direct interaction of its main components and the three targeted proteins, molecular docking studies were performed on the enzymes crystallographic structures retrieved from the Protein Data Bank (PDB codes: 3TOP, 4W93 and 1LPB for α-glucosidase, α-amylase and pancreatic lipase, respectively, Figure 1)^26–28^.

In 3TOP the protein is complexed with α-acarbose (AC1), while in 4W93 the pancreatic a-amylase is complex with montbretin A (3L9) and in 1LPB the enzyme binds colipase and two molecules of an alkilphosphonate inhibitor (MUP-A and MUP-B). In the first step of our simulation procedure, re-docking experiments have been performed in order to calculate the binding energy value for the crystallographic ligands for each target (Figure 2, panel D).

Figure 2.Ligand-binding pocket of the active site of α-glicosidase; ribbons representing protein structural elements are also shown. (A) Superimposed binding modes of all the twelve the ligands: AC1 (hotpink), caffeic acid (brown), ferulic acid (yellow), vanillic acid (magenta), homovanillic acid (orange), *p*-coumaric acid (pink), rosmarinic acid (blue), chlorogenic acid (green), coumarin (wheat), herniarin (bluewhite), apigenin (darksalmon), luteolin (deeppurple), quercetin (deepolive); the key residues are also indicated in the specific binding mode of (B) AC1; (C) caffeic acid; (D) ferulic acid; (E) vanillic acid; (F) homovanillic acid; (G) *p*-coumaric acid; (H) rosmarinic acid; (I) chlorogenic acid; (L) coumarin; (M) herniarin; (N) apigenin; (O) luteolin; (P) quercetin; (Q) Hot-spots identified where compounds accommodate when the main active site is occupied (for details, see supplementary material).

**Figure:**
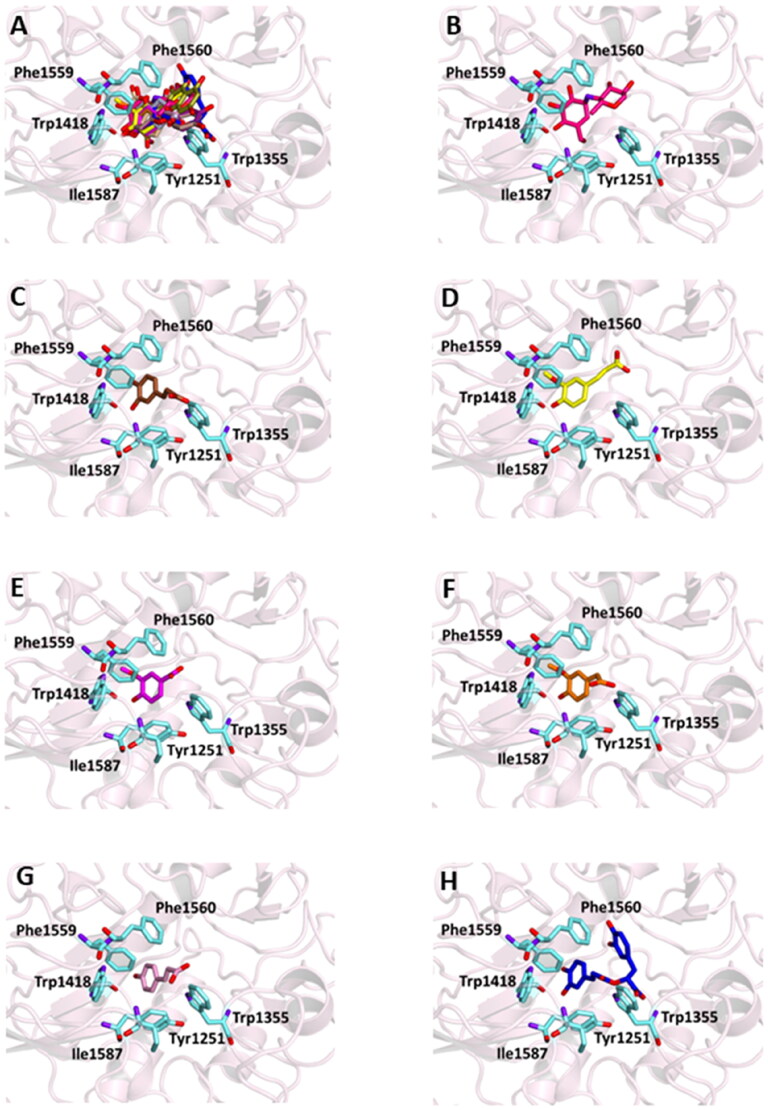

**Figure:**
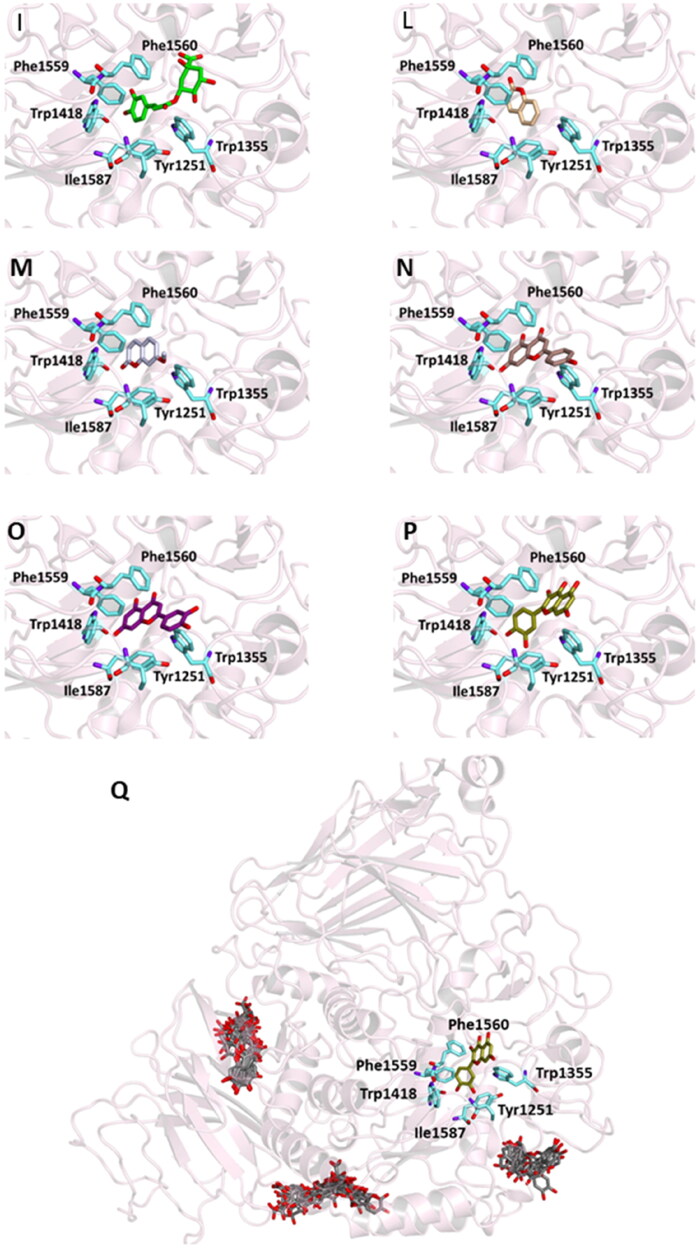

Successively, all the 12 main compounds identified in the extract were docked into the three enzymes to investigate their binding mode. In particular, the α-glucosidase (also known as maltase-glucoamylase, MGAM) is characterised by the presence in its structure of two catalytic sites on the amino- and carboxy-terminal domains, respectively.

The reaction catalysed by these portions of the enzyme, which show about 40% similarity, consists in hydrolysing α,1,4 linked maltose so producing glucose^70^. On the other hand, it has been proved that the carboxy-terminal active domain (CtMGAM) is able to bind larger substrates as it contains an additional 21 amino acid insert and has at least two more sub-sites in comparison with NtMGAM^26^^,^^70^.

In order to examine the interactions among the selected active substances and α-glucosidase, the compounds were docked with 3TOP, corresponding to CtMGAM, and their binding mode has been compared to that of the crystallographic ligand acarbose, that is also a well-known enzyme inhibitor.

As a results, the binding energy values obtained for all the compounds was comparable with that of acarbose. In particular, rosmarinic acid, chlorogenic acid, luteolin and quercetin showed lower free binding energy values (-8.9, −8.2, −8.2, −8.9 Kcal/mol, respectively) suggesting that these molecules could bind to glucosidase more readily than acarbose.

On the other hand, the score becomes less favourable when as the degrees of freedom of the molecules decrease down to the minimum value of −6 kCal/mol for coumarin. Almost all ligands were able to bind the enzyme interface similar to acarbose, establishing several hydrogen bonds with key residues of the active site and for all of them numerous hydrophobic and van der Waals contacts are involved in the ligand-target complex stabilisation. A detailed analysis of the docking results for the best pose of the molecules is presented in Table 5, while the best docking conformations are depicted in Figure 2.

**Table 5.** Chemical structures, dihedral angles, complexes binding energy values and key protein residues of α-glucosidase (3TOP) interacting with the ligands.

Ligand	Structure	Dihedral angles	Binding energy Kcal/mol	INTERACTIONS							
				Hydrogen Bonds				Hydrophobic Bonds Residues	π -stacking	Salt bridges	
				Residues	Distance Å		Donar Angle°				
					H-A	D-A					
Caffeic acid	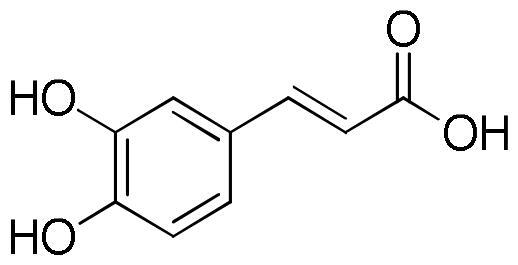	5	−6.8	Asp1279 Asp1420 Thr1586	2.49 2.18 2.38	3.06 2.97 2.79	117.31 137.49 105.06	Tyr1251 Trp1355 Trp1355 Phe1559	Phe1559		
Ferulic acid	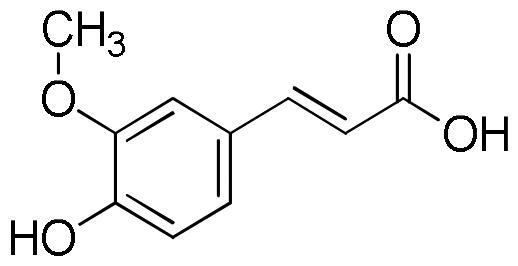	5	−7.1	His1584	2.12	3.07	164.92	Trp1355 Phe1559	Tyr1251		
Vanilic acid	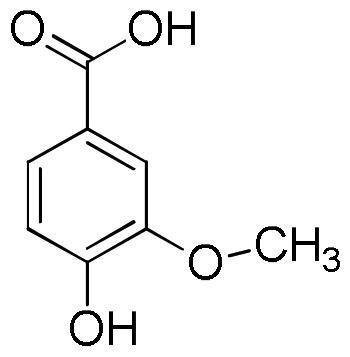	4	−6.3					Tyr1251 Tyr1251 Trp1355 Phe1559			
Homovanillic acid		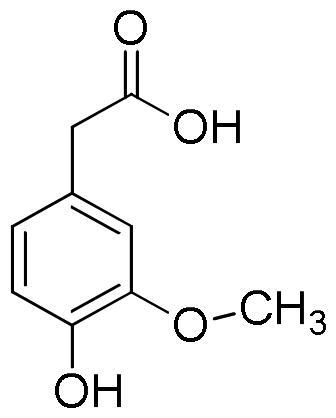	4	−6.7	Asp1279	2.16	3.07	155.26	Tyr1251 Tyr1251 Trp1355 Trp1355 Phe1559		
*p*-Coumaric acid	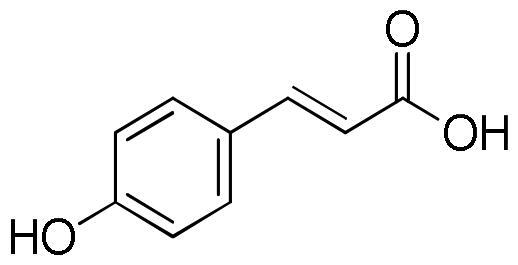	3	−6.4	Thr1586	2.35	2.89	116.38	Tyr1251 Trp1355 Trp1355 Phe1559 Phe1559	Tyr1251		
Rosmarinic acid	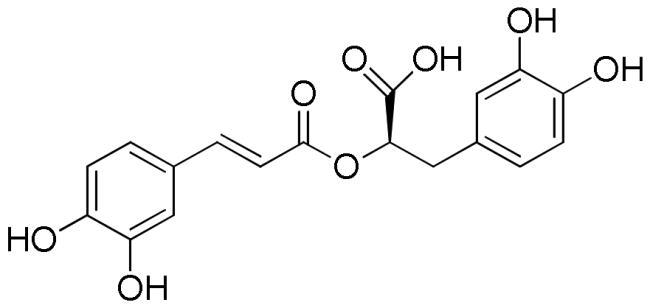	11	−8.9	Asp1279 Asp1420 Arg1510 Asp1526 Asp1526 Thr1586	2.50 2.28 2.72 2.01 2.69 2.47	2.98 2.88 3.70 2.82 3.58 3.01	110.06 121.89 171.46 137.95 152.81 115.11	Tyr1251 Trp1355 Phe1559 Phe1559 Phe1560 Phe1560	Tyr1251 Phe1559		
Chlorogenic acid	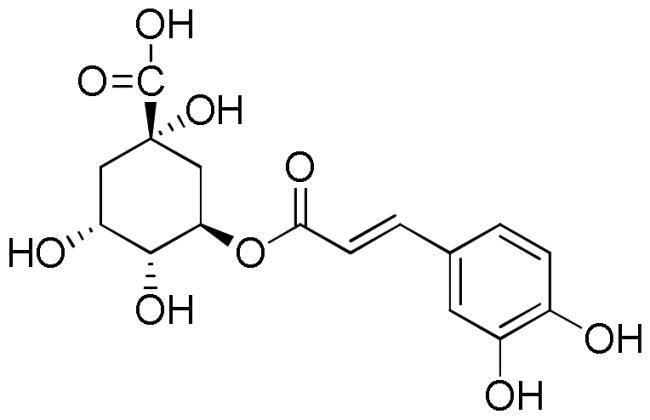	5	−8.2	Asp1420 Arg1510 Asp1526 Thr1586	2.98 2.62 3.51 2.91	3.68 3.05 3.82 3.59	133.45 107.06 100.81 119.35	Tyr1251 Trp1355 Trp1355 Phe1559	Tyr1251	Lys1460	
Coumarin	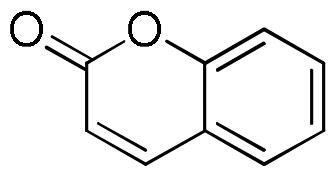	0	−6.0					Trp1355 Trp1418 Trp1523 Phe1559	Tyr1251	Arg1510	
Herniarin	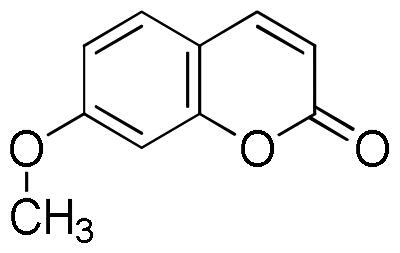	1	−6.3					Tyr1251 Trp1355 Phe1559		His1584	
Apigenin	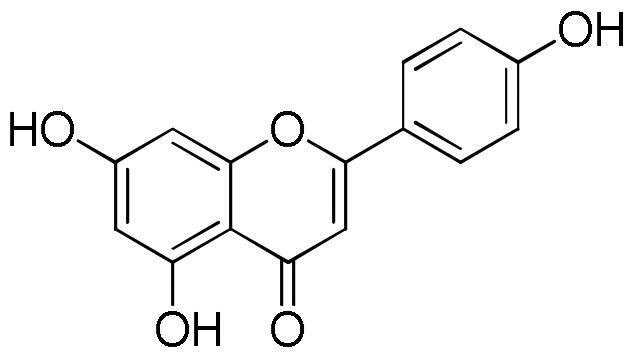	4	−8.1	Arg1510 Arg1510 Asp1526	1.99 3.49 3.33	2.92 4.09 4.02	156.50 121.42 130.00	Tyr1251 Trp1355 Ile1587	Trp1355 Phe1559		
Luteolin	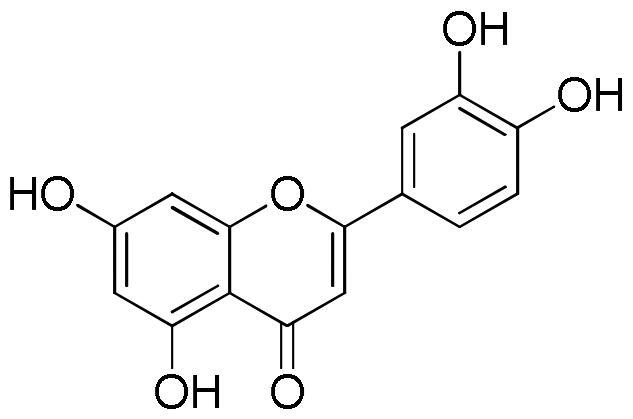	5	−8.2	Asp1420 Arg1510 Arg1510	3.46 1.91 3.48	3.83 2.83 4.06	104.31 153.56 119.34	Tyr1251 Trp1355 Ile1587	Trp1355 Phe1559		
Quercetin	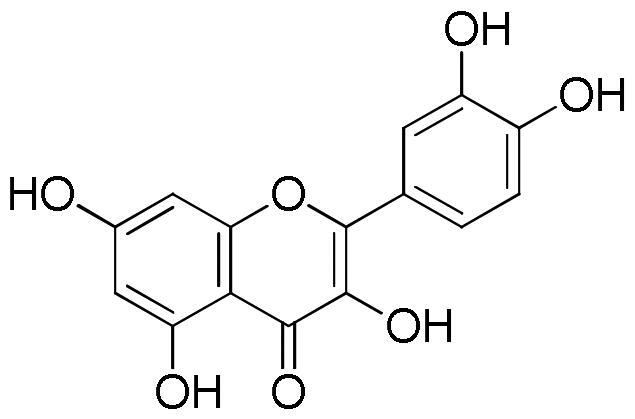	6	−8.9	Asp1157 Asp1279 Arg1510 Arg1510	2.80 2.24 2.06 3.26	3.75 2.99 3.04 3.95	176.07 133.18 172.78 128.69	Trp1355 Phe1559	Trp1355 Phe1560		

In order to establish if the better activity of the extract compared to the single component could be ascribed to a concomitant bind of more than one molecule to the enzyme, a blind docking protocol was applied. By a systematic exploration of the entire protein volume of 3TOP, three hot-spots where the ligands allocated when the ligand-binding site is occupied have been identified (Figure 2, panel Q).

In particular, the majority of the poses occupied the subsite surrounded by Tyr1618, Leu1622, Lys1625, Tyr1715, Trp1749, Val1631, Pro1658, albeit with slightly less favourable binding energy values than those obtained within the main catalytic site. These findings support the idea that compounds can have a synergistic action when simultaneously present and are consistent with those acquired during the *in vitro* studies. Table S2 and Figures S1 (Supplementary Materials) show the docking results for the best pose of the molecules in this secondary site.

The same protocol has been applied to investigate the binding mode of the selected phytochemicals to α-amylase, using 4W93. In this structure, the enzyme is complexes with montbretin A, as a potent known competitive and highly selective inhibitor. The compound structure includes a myricetin core functionalised with two carbohydrate chains at O3 of the benzopyrone and at C4 of the phenyl moiety, and a caffeic acid moiety attached to C6 of the first glucose in the chain^70^. Previous studies on this compound bonded to a-amylase demonstrated that myricetin alone is able to bind the enzyme active site whereas the sugars were not crucial for the binding^71^.

Since several of the selected compounds resemble the various portion of the crystallographic ligand, we perform our docking experiments to investigate the behaviour of each of them.

α-Amylase structure includes three distinct domains, and the active site is located to one end of a triosephosphate isomerase barrel. In our experiments (Table 6) all compounds occupied this binding site interacting with key residues essential for catalysis such us with binding energy values ranging from the best value of −8.9 kcal/mol for luteolin and quercetin to the less favourable −5.4 kcal/mol value for coumarin.

**Table 6.** Complexes binding energy values and key protein residues of α-amylase (4W93) interacting with the ligands.

Ligand	Binding Energy Kcal/Mol	INTERACTIONS						
		Hydrogen Bonds				Hydrophobic Bonds Residues	π-stacking	Salt Bridges
		Residues	Distance Å		Donar Angle°			
			H-A	D-A				
Caffeic acid	−6.5	Gln63 Arg195 Arg195 Glu233	2.07 3.30 2.15 2.96	2.99 4.00 3.12 3.91	155.49 129.64 167.47 168.34	Trp58 Tyr62 Leu165	Tyr62	
Ferulic acid	−6.5	Gln63 Arg195 Arg195 Glu233	2.08 3.35 2.14 2.59	2.99 4.04 3.11 3.00	153.71 129.06 168.24 105.82	Trp58 Tyr62 Leu165	Tyr62	
Vanilic acid	−5.5	Tyr62 Arg195 Arg195 Ala198	3.52 3.30 2.09 3.53	4.09 3.98 3.06 3.94	121.52 128.38 167.42 107.51	Trp58 Tyr62	Tyr62	
Homovanillic acid	−5.8	Trp59 Arg195 Arg195 Ala198 Glu233	3.41 3.36 2.15 3.70 2.31	4.06 4.06 3.12 4.08 3.00	128.30 128.99 167.90 105.95 127.32	Trp58 Trp59 Tyr62	Tyr62	
*p*-Coumaric acid	−6.2	Gln63 Arg195 Arg195 Glu233	2.02 3.18 2.39 3.47	2.92 3.89 3.27 3.83	151.32 130.13 148.62 104.78	Trp58 Tyr62 Leu165	Tyr62	
Rosmarinic acid	−7.9	Trp59 Tyr62 Gln63 Arg195 Arg195 Ala198 Glu233 His299	3.08 3.55 2.43 2.65 2.23 3.64 2.29 3.04	3.40 4.10 3.33 3.34 3.02 4.00 3.03 3.76	101.91 117.70 151.73 127.41 135.90 103.91 132.34 132.18	Trp58 Trp58 Trp59 Leu162 Ala198 Asp300	Tyr62	His101
Chlorogenic acid	−7.4	Gln63 Asn105 Ala106 Thr163 Thr163 Gly164	3.36 2.69 2.37 3.30 1.98 3.04	3.99 3.14 3.30 3.93 2.88 3.43	123.76 107.83 157.56 124.19 152.95 106.70	Tyr62		
Coumarin	−5.4	Gln63	2.36	3.27	153.26	Trp58 Tyr62 Leu165	Tyr62	
Herniarin	−5.9	Gln63	2.35	3.27	154.08	Trp58 Tyr62 Leu165	Tyr62	
Apigenin	−8.6	Trp59 Gln63 Arg195 Arg195 Glu233	2.96 2.35 3.05 2.39 3.06	3.67 2.90 3.81 3.30 3.95	130.51 114.15 134.62 153.18 153.79	Trp58 Tyr62	Phe215 His263	
Luteolin	−8.9	Gln63 Arg195 Arg195 Asp197 His299	2.30 3.06 2.35 2.13 2.73	2.87 3.81 3.26 3.07 3.66	115.57 133.72 153.83 166.68 163.26	Trp58 Tyr62	Trp59 Trp59 Tyr62	
Quercetin	−8.9	Gln63 His101 Arg195 Arg195 Asp197 Ala198	2.27 3.48 3.10 2.02 2.32 3.60	2.88 3.97 3.81 2.99 2.77 3.98	118.49 114.44 127.74 167.39 107.24 105.57	Trp58 Trp59 Trp59 Tyr62	Trp59 Tyr62	

This could be explained by the reduced possibility that the compound forms the same number of hydrogen bonds as other phytochemicals that have numerous hydroxyl groups in their structure. In particular, six out of the compounds form a hydrogen bond with Glu233 and, as demonstrated by previous studies, this interaction precludes its role in the catalytic function as an acid-base promoter of hydrolysis, while luteolin and quercetin were able to interact with Asp197, a crucial residue for the nucleophilic α-amylase hydrolytic catalysis on starch polymers^72^^,^^73^. For all compounds, several hydrophobic interactions contribute to stabilise the complexes. A detailed analysis of the docking results for the best pose of the molecules is presented in Table 6, while the best docking conformations are depicted in Figure 3.

Figure 3.Ligand-binding pocket of the active site of α-amylase; ribbons representing protein structural elements are also shown. (A) Superimposed binding modes of all the twelve the ligands: 3L9 (hotpink), caffeic acid (brown), ferulic acid (yellow), vanillic acid (magenta), homovanillic acid (orange), *p*-coumaric acid (pink), rosmarinic acid (blue), chlorogenic acid (green), cumarin (wheat), erniarin (bluewhite), apigenin (darksalmon), luteolin (deeppurple), quercetin (deepolive); the key residues are also indicated in the specific binding mode of (B) 3L9; (C) caffeic acid; (D) ferulic acid; (E) vanillic acid; (F) homovanillic acid; (G) *p*-coumaric acid; (H) rosmarinic acid; (I) chlorogenic acid; (L) coumarin; (M) herniarin; (N) apigenin; (O) luteolin; (P) quercetin; (Q) Hot-spots identified where compounds accommodate when the main active site is occupied (for details, see supplementary material).

**Figure:**
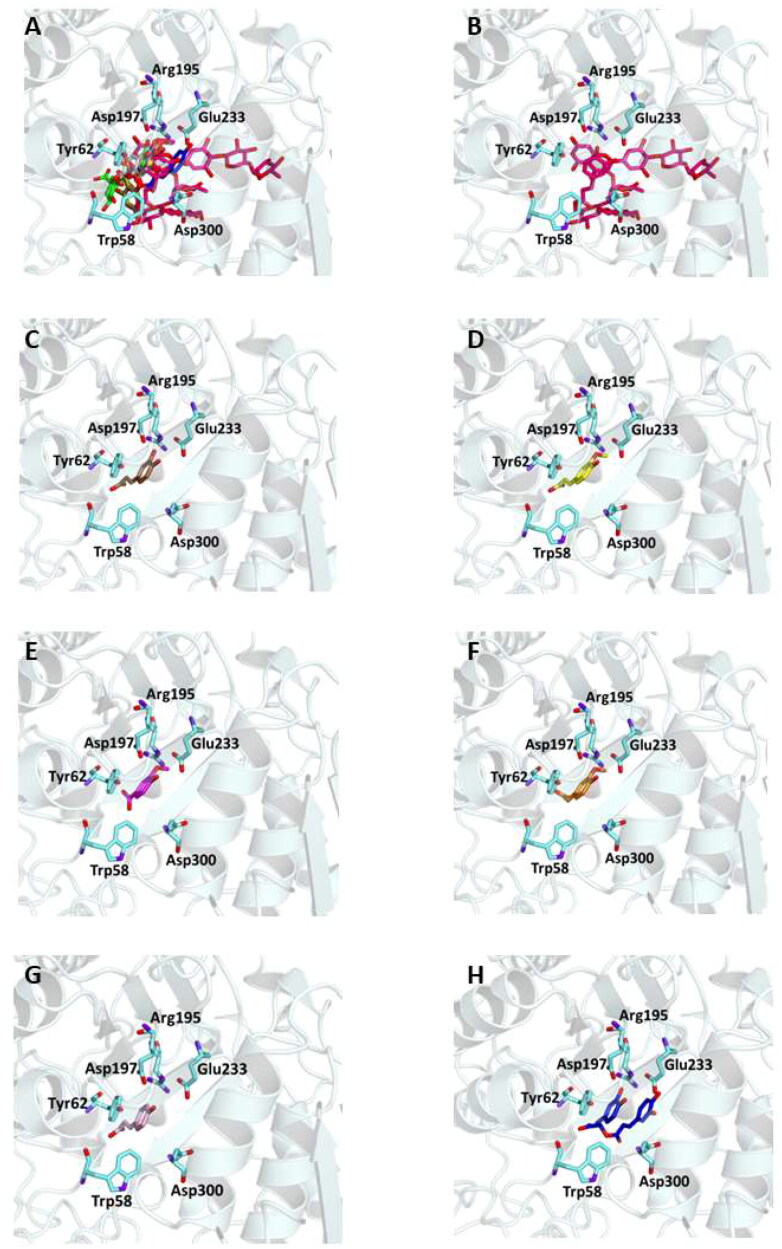

**Figure:**
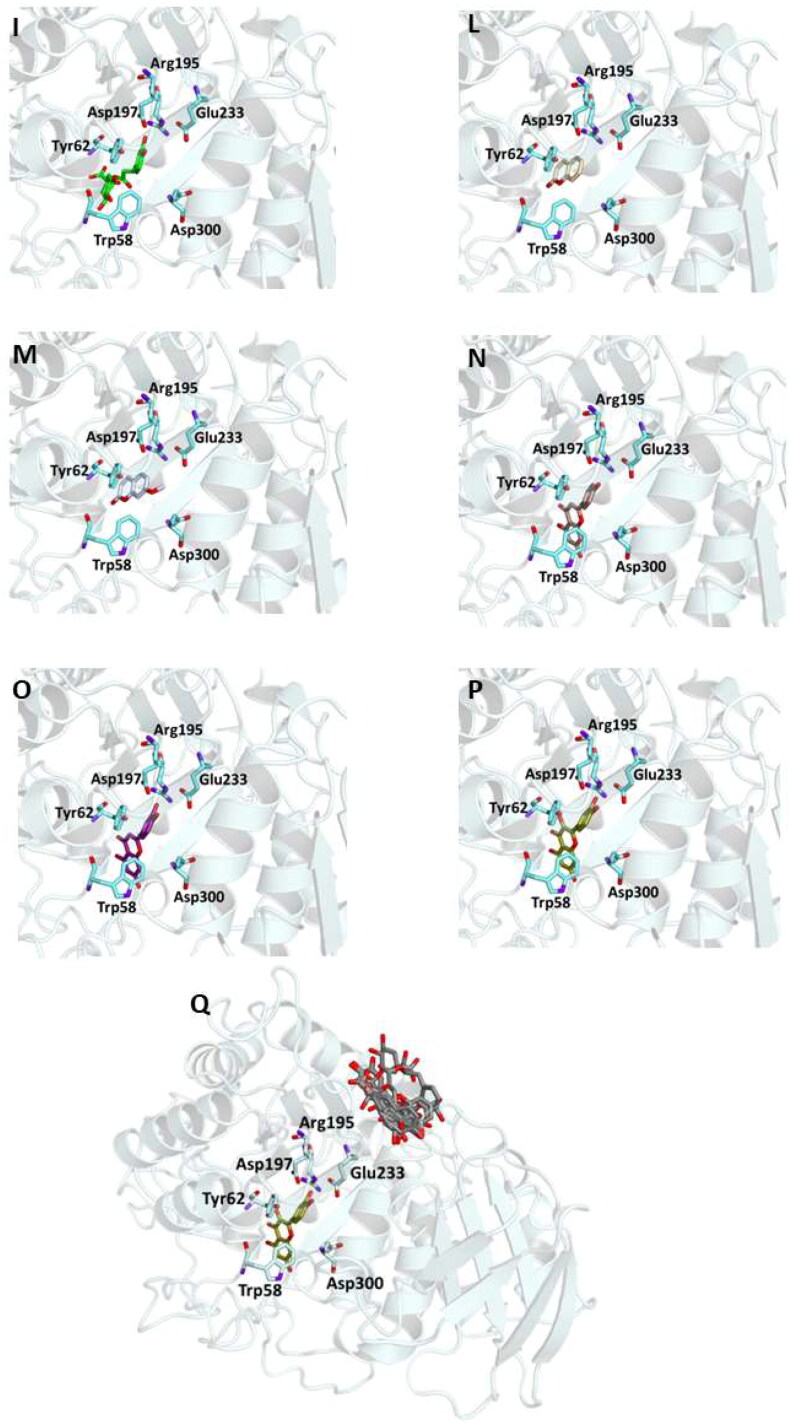

Also in this case, to support the biological activity recorded for the plant extract, blind docking was performed and after a systematic exploration of the entire protein volume of 4W93 a subsite in which the majority of compound poses accommodate when the main active site is occupied has been identified (Figure 3, panel Q). These results suggest that the subsite could act as an allosteric site contributing to the catalytic activity of a mixture of active compounds (Table S3 and Figure S2).

Similar to the studies carried out on 3TOP and 4W93, the modalities of interaction between the twelve compounds and the pancreatic lipase were investigated by means of docking procedures using the PDB entry 1LPB, in which the enzyme is crystallised with two molecules of a previously identified a C11 alkylphosphonate inhibitor (MUP).

The enzyme structure includes three main domains: a non-catalytic C-terminus containing a colipase binding site, a *N*-terminal domain including the active site characterised by a Ser152-Asp176-Hys263 catalytic triad, and a lid loop that modulates the ligand entry into the active site. All tested compounds are able to bind the active site in a similar manner of the crystallographic ligand interacting with key residues involved in lipid hydrolysis (Figure 4, Table 7).

Figure 4.Ligand-binding pocket of the active site of the active site of pancreatic lipase; ribbons representing protein structural elements are also shown. (A) Superimposed binding modes of all the twelve the ligands: MUP (hotpink), caffeic acid (brown), ferulic acid (yellow), vanillic acid (magenta), homovanillic acid (orange), *p*-coumaric acid (pink), rosmarinic acid (blue), chlorogenic acid (green), coumarin (wheat), erniarin (bluewhite), apigenin (darksalmon), luteolin (deeppurple), quercetin (deepolive); the key residues are also indicated in the specific binding mode of (B) MUP; (C) caffeic acid; (D) ferulic acid; (E) vanillic acid; (F) homovanillic acid; (G) *p*-coumaric acid; (H) rosmarinic acid; (I) chlorogenic acid; (L) coumarin; (M) herniarin; (N) apigenin; (O) luteolin; (P) quercetin; (Q) Hot-spots identified where compounds accommodate when the main active site is occupied (for details, see supplementary materials).

**Figure:**
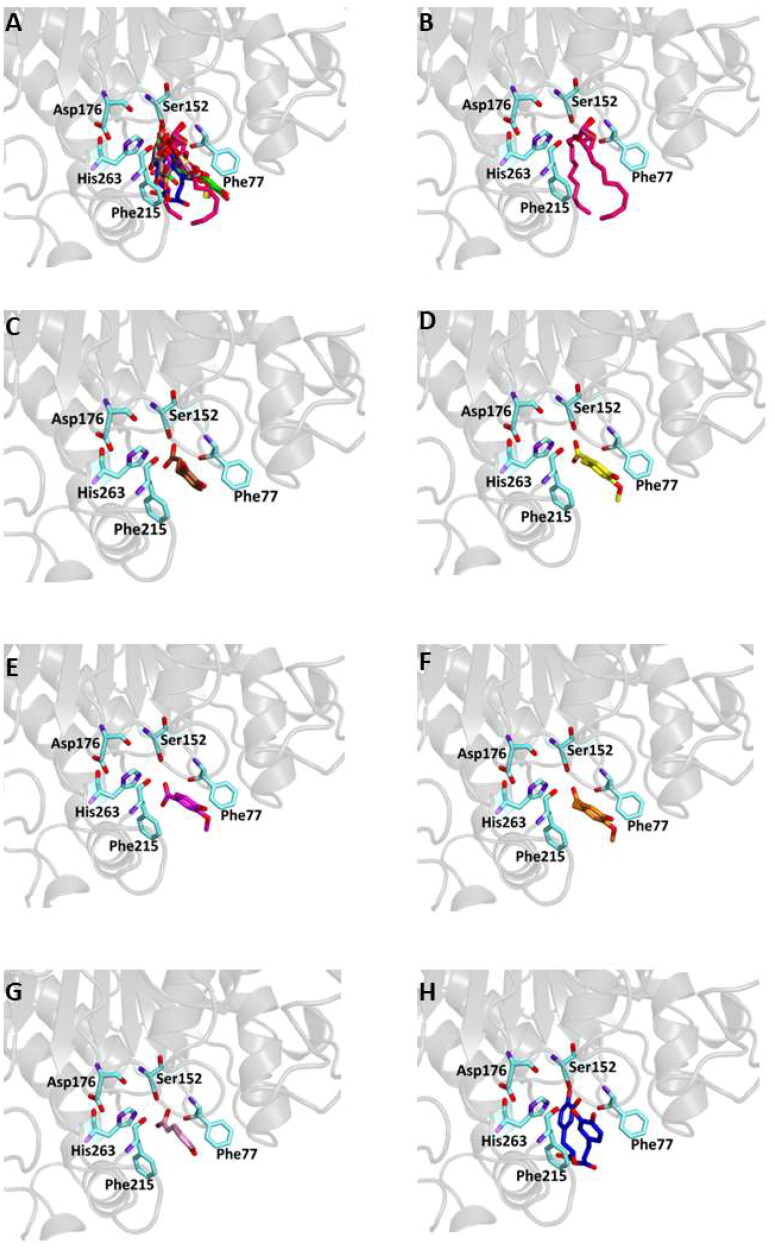

**Figure:**
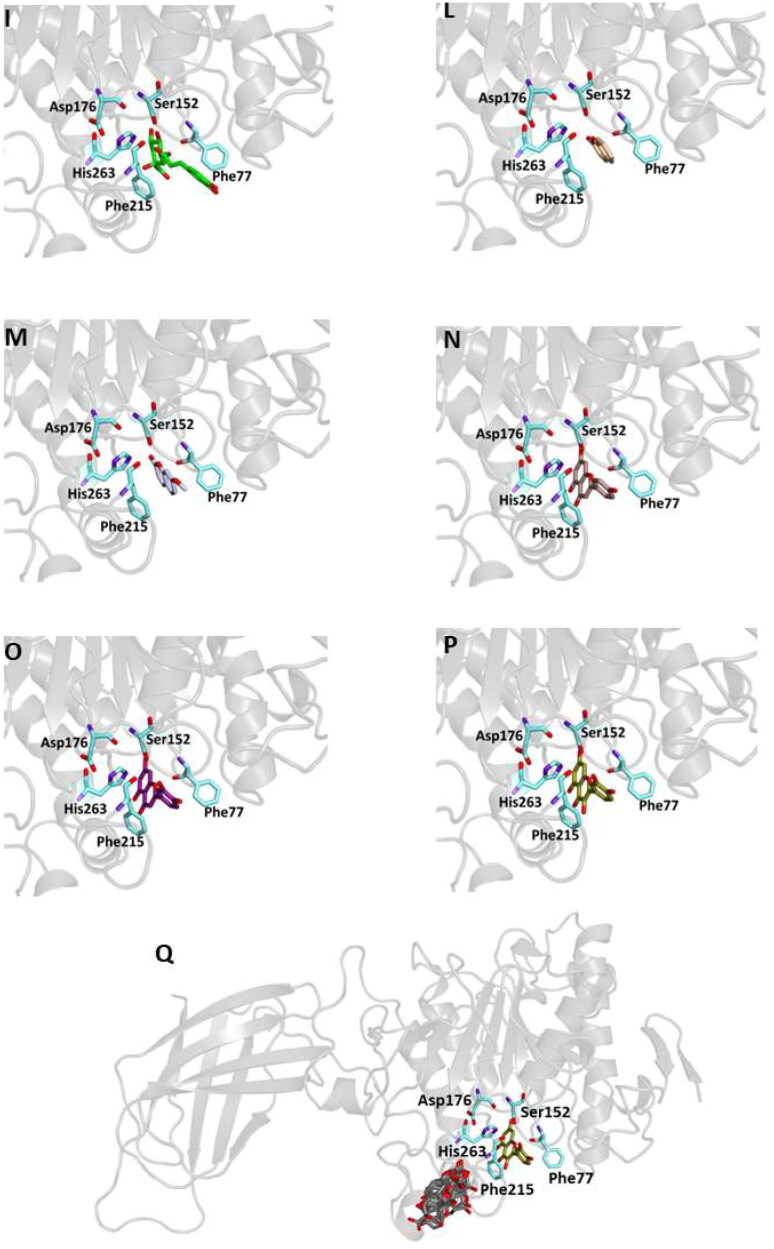

**Table 7.** Binding energies for and key protein residues of lipase receptor (1LPB) interacting with the ligands.

Ligand	Binding energy Kcal/mol	Interactions						
		Hydrogen bonds				Hydrophobic bonds residues	π-stacking	Salt Bridges
		Residues	Distance Å		Donar Angle°			
			H-A	D-A				
Caffeic acid	−6.3	Gly76 Tyr114 Tyr114 Glu179	3.41 3.65 3.15 3.23	3.80 4.01 4.01 4.02	106.40 105.90 148.46 137.67	Phe77 Phe77 Phe215	Phe215	His151 His263
Ferulic acid	−6.1	Phe77 Ser152 Ser152	3.43 2.48 2.45	4.00 3.25 3.25	118.54 135.79 142.76	Phe77 Phe77 Tyr114 Pro180 Phe215		His151 His263
Vanilic acid	−5.6	Ser152 Ser152	1.88 2.16	2.76 2.76	149.59 120.74	Phe77 Tyr114 Pro180 Phe215		His263
Homovanillic acid	−5.8	Ser152	2.04	2.97	161.11	Phe77 Tyr114 Pro180 Phe215 Phe215		His263
*p*-Coumaric acid	−6.2	Ser152 Ser152	3.40 3.64	4.10 4.10	131.00 111.17	Phe77 Phe77 Tyr114 Tyr114 Phe215 Phe215		His151 His263
Rosmarinic acid	−8.4	Gly76 Phe77 Phe77 Asp79 Tyr114 His151 Ser152 Ser152	2.53 2.60 2.53 2.76 2.44 2.46 1.87 2.91	3.27 3.07 3.28 3.13 3.22 3.24 2.70 3.67	132.26 109.81 133.89 104.19 136.64 135.64 141.82 135.57	Phe77 Tyr114 Phe215 Phe215 Ala260 Leu264	Phe215	
Chlorogenic acid	−8.8	Gly76 Phe77 His151 Ser152	3.13 3.21 2.55 2.17	3.90 3.77 3.21 2.98	136.11 118.28 123.97 140.76	Phe77 Phe77 Tyr114 Pro180 Phe215	Tyr114	Arg256 His263
Coumarin	−7.0	Ser152	2.90	3.81	157.12	Phe77 Ala178 Pro180 Phe215	Phe215 Phe215	His263
Herniarin	−6.7	Phe77 Ser152	3.50 2.95	3.96 3.69	110.94 133.24	Phe77	Phe215 Phe215	His151 His263
Apigenin	−9.3	Gly76 Asp79 His151	2.29 2.56 2.23	3.09 3.01 3.06	137.06 110.30 141.92	Phe77 Phe215 Leu264	Phe215 His263	
Luteolin	−9.6	Gly76 Phe77 Asp79 Tyr114 Tyr114 Tyr114 His151 Glu179	2.33 3.49 2.52 3.61 3.21 3.28 2.24 3.25	3.13 3.94 2.97 3.94 3.94 4.04 3.07 4.03	138.00 110.39 109.52 103.79 133.15 136.71 141.49 137.19	Phe77 Phe215 Leu264	Phe215 His263	
Quercetin	−9.3	Gly76 Asp79 Asp79 Tyr114 His151 Phe215	2.25 2.48 2.56 3.69 2.15 2.44	3.07 2.96 2.96 4.06 3.00 2.94	139.41 112.22 104.40 106.85 143.85 111.38	Phe77 Phe215 Leu264	Phe215 His263	

From our data, it seems that apigenin, luteolin and quercetin form the most stable complexes with 1LPB, as a results from their favourable binding energy values (−9.3, −9.6 and −9.3 kcal/mol, respectively). Out of hydrogen bonds, hydrophobic and van der Waals contacts seems to play a key role in the ligand-target complex stabilisation for all the studied compounds.

A putative allosteric binding site has been identified also in 1LPB, after blind docking by systematic exploration of the entire protein volume. In fact, the majority of the poses of all the 12 compounds accommodate into this subsite when the active site is occupied. Furthermore, it is very close to the main binding site, so an allosteric modulation of the catalytic activity due to the binding of more than one compound to lipase is plausible. Table S4 and Figure S3 show the docking results for the best pose of the molecules in this secondary site.

The overall results of docking studies provide evidence of the capability of the main *L. angustifolia* extract compounds to interact with key residues of the active sites of the three targets with favourable binding energy, thus suggesting that they could represent suitable leads for the development of novel tools for the treatment of metabolic disorders.

Furthermore, in all cases one or more hot-spots where compounds accommodate when the main active site is occupied have been identified so hypothesising a concomitant bind of more than one compound to the protein that could justify the higher activity of the entire extract compared to each single compounds resulting from biological studies.

## Conclusions

Controlling the response of carbohydrate hydrolysing enzymes (α-glycosidase and α-amylase) and pancreatic lipase is an effective approach in treating post-prandial hyperglycaemia and fats metabolism in patients affected by TD2 and obesity. In recent years, inhibitors of these enzymes have received great attention from researchers, and inhibitors from natural source have still attracted much attention due to their wide range of sources, structural diversity, and low toxicity and side effects.

The results of this study showed that *L. angustifolia* ethanolic extracts have significant inhibitory activities against these enzymes, better than the positive controls acarbose and orlistat.

These effects are consistent with the results of molecular docking studies conducted on the crystallographic structures of enzymes. Indeed, our experiments confirm that the most abundant compounds of lavender extracts are sterically compatible with the accommodation within the active site of target proteins occupied by known ligands.

The calculated favourable binding energy values support the formation of stable complexes. Furthermore, the identification of hot spots outside the catalytic site suggests a potential synergistic action of compounds that could simultaneously bind the targets.

Overall, the encouraging results obtained in this study highlight the potentiality of lavender ethanolic extracts and their main constituents as promising candidates for the development of new nutraceuticals, useful as adjuvants for the prevention and/or treatment of metabolic disorders.

However, more detailed *in vivo* studies will be needed to establish therapeutic dosage, upcoming specific clinical formulations, and ways of administration, being clinical trials essential to deepen knowledge through. Additionally, the isolation of the active constituents will be of interest for developing into a new and effective antidiabetic drug.

## Supplementary Material

Supplemental Material
