# Supplementary material for: Lavandula angustifolia mill. (Lamiaceae) ethanol extract and its main constituents as promising agents for the treatment of metabolic disorders: chemical profile, in vitro biological studies, and molecular docking
Source: J Enzyme Inhib Med Chem. 2023 Oct 18;38(1):2269481. doi: 10.1080/14756366.2023.2269481 (PMC10586085; doi:10.1080/14756366.2023.2269481)
Supplement: Supplemental Material [file IENZ_A_2269481_SM9785.pdf]

Supplementary material

CONTENTS

Table S1. Limit of detection (LOD), limit of quantification (LOQ) and regression.

Table S2. Binding energy values and protein residues involved in the interactions with the selected α-glucosidase secondary site.

Figure S1. Binding modes of identified compounds and the selected α-glucosidase secondary site.

Table S3. Binding energy values and protein residues involved in the interactions with α-amylase secondary site.

Figure S2. Binding modes of identified compounds and α-amylase secondary site.

Table S4. Binding energy values and protein residues involved in the interactions with pancreatic lipase secondary site.

Figure S3. Binding modes of identified compounds and pancreatic lipase secondary site

Table S1. Limit of detection (LOD), limit of quantification (LOQ) and regression.

Compounds	Regression equation	R^2	LOD µg/g	LOQ µg/g
**Coumarin**	y = 44.25x – 50.09	0.9998	0.0466	0.3143
**Chlorogenic acid**	y = 53.15x – 99.73	0.9999	0.0532	0.2965
**Caffeic acid**	y = 113.33x – 109.33	0.9997	0.0782	0.4652
***p*-Coumaric acid**	y= 115.96x + 52.26	0.9999	0.0988	0.1987
**Homovanillic acid**	y= 60.097x + 120.01	0.9999	0.0456	0.2354
**Quercetin**	y = 47.63x + 53.21	0.9996	0.0128	0.2465
**Luteolin**	y= 74.58x – 136.08	0.9996	0.0234	0.4653
**Apigenin**	y= 101.81x – 300.00	0.9999	0.0355	0.3456
**Herniarin**	y= 46.07x – 14.44	0.9995	0.04536	0.2176
**Ferulic acid**	y= 76.663x – 124.87	0.9994	0.0324	0.1682
**Rosmarinic acid**	y= 228.72x – 194.39	0.9997	0.1123	0.4521
**Vanillic acid**	y= 81.439x – 1.2768	0.9997	0.1423	0.3521

Table S2. Complexes binding energy values and protein residues of one of the α-glycosidase secondary site interacting with the ligands when the main binding site is occupied. (PDB entry: 3TOP) The choice of this subsite has been made in consideration of literature data and the high number of compounds' poses in the hotspot.

LIGAND	Binding Energy Kcal/mol	INTERACTIONS						
		Hydrogen Bonds				Hydrophobic Bonds Residues	π-stacking	Salt Bridges
		Residues	Distance Å H-A	Distance Å D-A	Donar Angle °			
**Caffeic acid**	-7.1	Thr1621<br>Glu1629<br>Trp1749	2.12<br>3.15<br>2.08	2.56<br>3.63<br>3.05	105.92<br>113.88<br>165.63	Pro1658<br>Tyr1715<br>Trp1749	Trp1749	Arg1635
**Ferulic acid**	-7.1	Glu1629<br>Trp1749	3.00<br>2.19	3.32<br>3.13	101.69<br>160.31	Leu1622<br>Pro1658<br>Trp1749	Tyr1618	
**Vanilic acid**	-6.0	Thr1621<br>Trp1749	2.43<br>2.49	2.80<br>3.23	102.46<br>132.24	Leu1622<br>Lys1625<br>Pro1658<br>Trp1749	Trp1749	Arg1635
**Homovanillic acid**	-6.6	Trp1749	2.51	3.22	128.94	Leu1622<br>Lys1625<br>Trp1749	Trp1749	Arg1635
***p*-Coumaric acid**	-7.1	Ile1716<br>Trp1749	2.53<br>2.10	2.89<br>3.07	101.91<br>167.76	Tyr1618<br>Pro1658<br>Tyr1715<br>Trp1749	Trp1749	Arg1635<br>Arg1635
**Rosmarinic acid**	-8.2	Ile1716<br>Ile1716<br>Trp1749<br>Phe1771	3.43<br>2.12<br>2.12<br>3.21	3.90<br>2.97<br>3.09<br>3.82	111.98<br>144.95<br>166.01<br>122.11	Tyr1618<br>Pro1658<br>Tyr1715<br>Tyr1715<br>Trp1749	Trp1749	Arg1635
**Chlorogenic acid**	-8.4	Arg1635<br>Ile1716<br>Ile1716<br>Trp1749	2.64<br>3.61<br>1.88<br>2.10	2.10<br>4.05<br>2.79<br>3.08	108.80<br>109.66<br>162.41<br>171.04	Leu1622<br>Lys1625<br>Pro1658<br>Trp1749	Tyr1618<br>Trp1749	
**Herniarin**	-6.6	Trp1749	2.47	3.07	118.67	Leu1622<br>Lys1625<br>Val1631<br>Pro1658<br>Trp1749	Trp1749	Arg1635
**Apigenin**	-9.1	Thr1621<br>Ile1716<br>Gly1747<br>Trp1749<br>Trp1749	2.22<br>2.97<br>2.62<br>3.27<br>2.01	2.92<br>3.37<br>3.10<br>3.91<br>2.72	128.69<br>105.30<br>112.84<br>123.93<br>128.69	Tyr1618<br>Lys1625<br>Pro1658<br>Tyr1715<br>Trp1749		
**Luteolin**	-9.3	Thr1621<br>Arg1635<br>Ile1716<br>Gly1747<br>Trp1749	2.28<br>2.28<br>2.97<br>2.62<br>3.28	2.99<br>2.95<br>3.37<br>3.11<br>3.91	128.67<br>124.45<br>105.49<br>114.17<br>123.00	Try1618<br>Lys1625<br>Val1631<br>Pro1658<br>Tyr1715<br>Trp1749	Trp1749	
**Quercetin**	-8.3	Thr1621<br>Tyr1715<br>Ile1716<br>Trp1749	2.16<br>3.65<br>2.33<br>1.95	2.95<br>4.03<br>2.98<br>2.88	138.13<br>106.16<br>126.15<br>157.07	Tyr1618<br>Pro1658<br>Pro1658<br>Trp1749	Tyr1715<br>Trp1749<br>Trp1749	

Figure S1

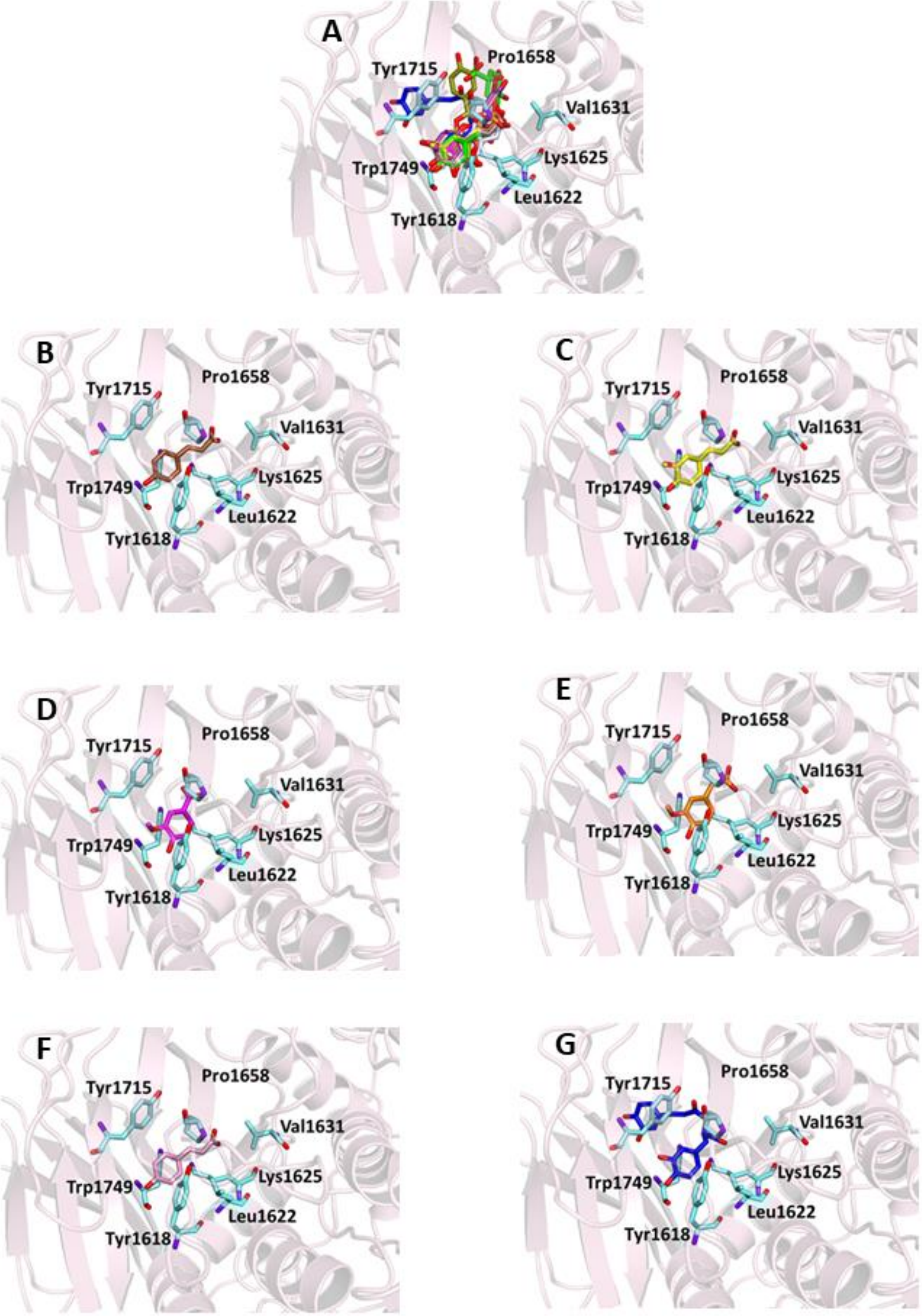
A
Tyr1715
Pro1658
Val1631
Lys1625
Trp1749
Leu1622
Tyr1618
B
Tyr1715
Pro1658
Val1631
Lys1625
Trp1749
Leu1622
Tyr1618
C
Tyr1715
Pro1658
Val1631
Lys1625
Trp1749
Leu1622
Tyr1618
D
Tyr1715
Pro1658
Val1631
Lys1625
Trp1749
Leu1622
Tyr1618
E
Tyr1715
Pro1658
Val1631
Lys1625
Trp1749
Leu1622
Tyr1618
F
Tyr1715
Pro1658
Val1631
Lys1625
Trp1749
Leu1622
Tyr1618
G
Tyr1715
Pro1658
Val1631
Lys1625
Trp1749
Leu1622
Tyr1618

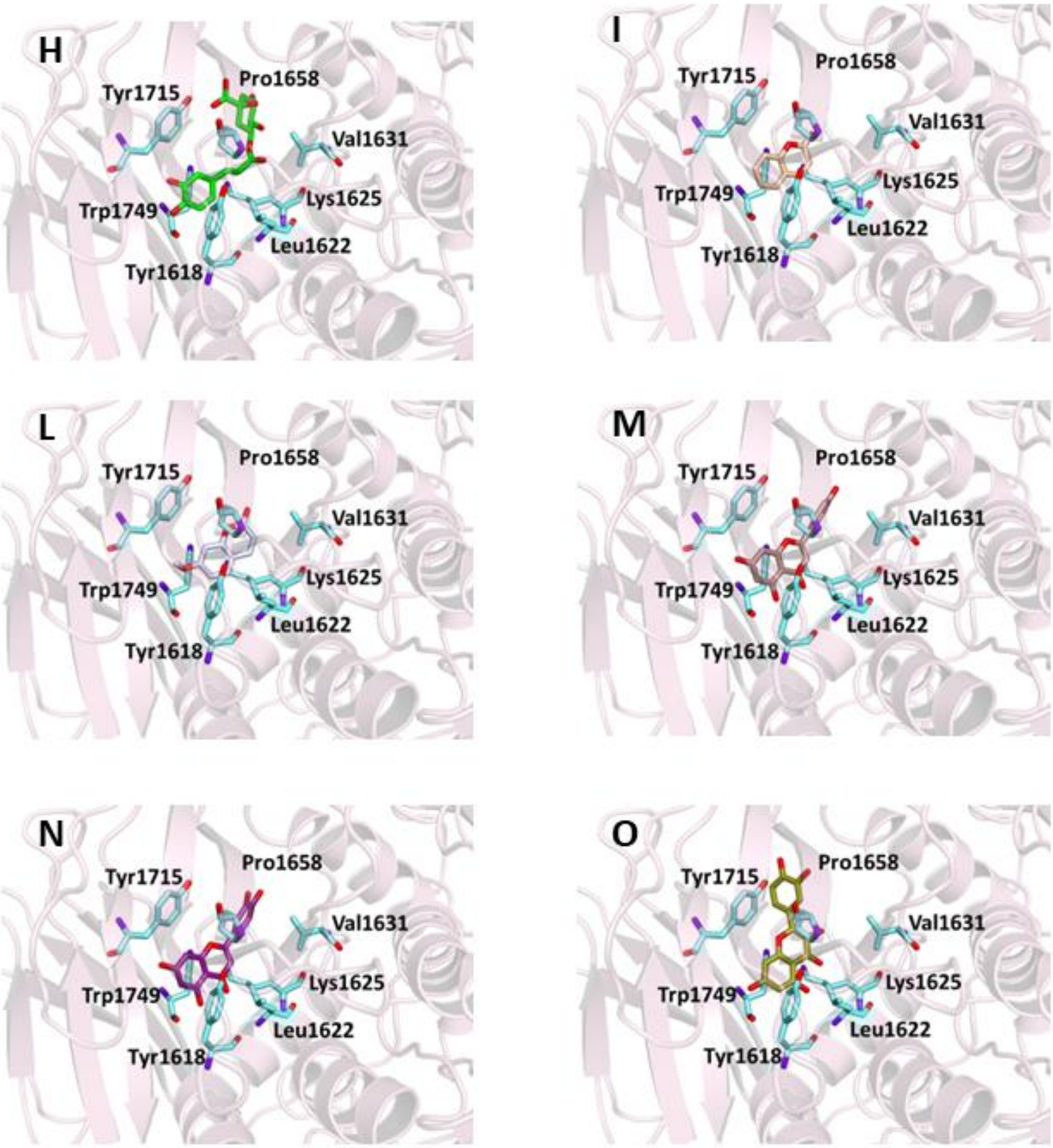

Ligand-binding pocket of the selected secondary site of α-glucosidase; ribbons representing protein structural elements are also shown. (**A**) Superimposed binding modes of all the twelve the ligands: caffeic acid (brown), ferulic acid (yellow), vanillic acid (magenta), homovanillic acid (orange), *p*-coumaric acid (pink), rosmarinic acid (blue), chlorogenic acid (green), coumarin (wheat), herniarin (blue-white), apigenin (dark-salmon), luteolin (deep-purple), quercetin (deep-olive); the interaction residues are also indicated in the specific binding mode of (**B**) caffeic acid; (**C**) ferulic acid; (**D**) vanillic acid; (**E**) homovanillic acid; (**F**) *p*-coumaric acid; (**G**) rosmarinic acid; (**H**) chlorogenic acid; (**I**) coumarin; (**L**) herniarin; (**M**) apigenin; (**N**) luteolin; (**O**) quercetin.

Table S3. Complexes binding energy values and protein residues of α-amylase secondary site interacting with the ligands (PDB entry: 4W93).

LIGAND	Binding Energy Kcal/mol	INTERACTIONS						
		Hydrogen Bonds				Hydrophobic Bonds Residues	π -stacking	Salt Bridges
		Residues	Distance Å		Donar Angle °			
			H-A	D-A				
**Caffeic acid**	-5.4	Thr6	3.14	3.82	126.88	Pro4<br>Thr11	Phe335	
		Arg252	3.05	3.81	134.87			
		Arg252	3.28	4.00	131.62			
		Arg398	2.06	2.90	141.83			
**Ferulic acid**	-5.2	Thr6	2.60	3.15	116.22	Pro4<br>Phe335		
		Arg252	2.29	3.19	150.93			
		Arg252	2.34	3.22	149.01			
		Ser289	2.67	3.19	114.63			
		Gly334	2.85	3.30	108.92			
**Vanilic acid**	-4.9	Thr6	3.40	4.00	121.81	Thr11<br>Phe335		
		Arg398	2.31	2.90	117.76			
		Arg398	3.09	4.00	157.39			
**Homovanillic acid**	-4.9	Pro332	2.68	3.06	103.68	Pro4<br>Thr11<br>Phe335		
		Arg398	2.34	2.94	117.92			
***p*-Coumaric acid**	-5.1	Thr6	3.15	3.81	125.81	Pro4<br>Thr11	Phe335	
		Arg398	2.12	2.96	142.61			
		Arg398	3.00	3.32	100.39			
**Rosmarinic acid**	-5.6	Thr11	2.61	3.11	111.96	Thr11<br>Asp290<br>Phe335		Arg252
		Arg252	2.83	3.81	173.75			
		Ser289	2.57	3.16	118.67			
		Ser289	2.17	2.71	113.31			
		Arg291	3.30	3.87	119.22			
		Pro332	2.47	2.82	100.48			
		Gly334	2.80	3.39	118.52			
		Gly334	1.87	2.73	147.07			
		Arg398	2.59	3.37	136.50			
**Chlorogenic acid**	-6.1	Ser3	2.47	3.24	134.75	Pro4<br>Phe335		Arg252
		Thr11	2.81	3.59	137.36			
		Arg252	1.95	2.80	143.45			
		Arg398	2.36	3.18	139.94			
**Coumarin**	-5.1					Pro4<br>Thr11	Phe335	Arg252
**Herniarin**	-5.2						Phe335	Arg252
**Apigenin**	-6.4	Ser289	2.47	3.33	147.78	Pro4		
		Gly334	1.95	2.83	149.57			
		Arg398	2.60	3.23	121.92			
**Luteolin**	-6.5	Arg398	2.58	3.22	122.66	Pro4		
**Quercetin**	-6.1	Ser3	2.62	3.07	108.63	Pro4		
		Arg398	3.25	4.00	134.26			

Figure S2

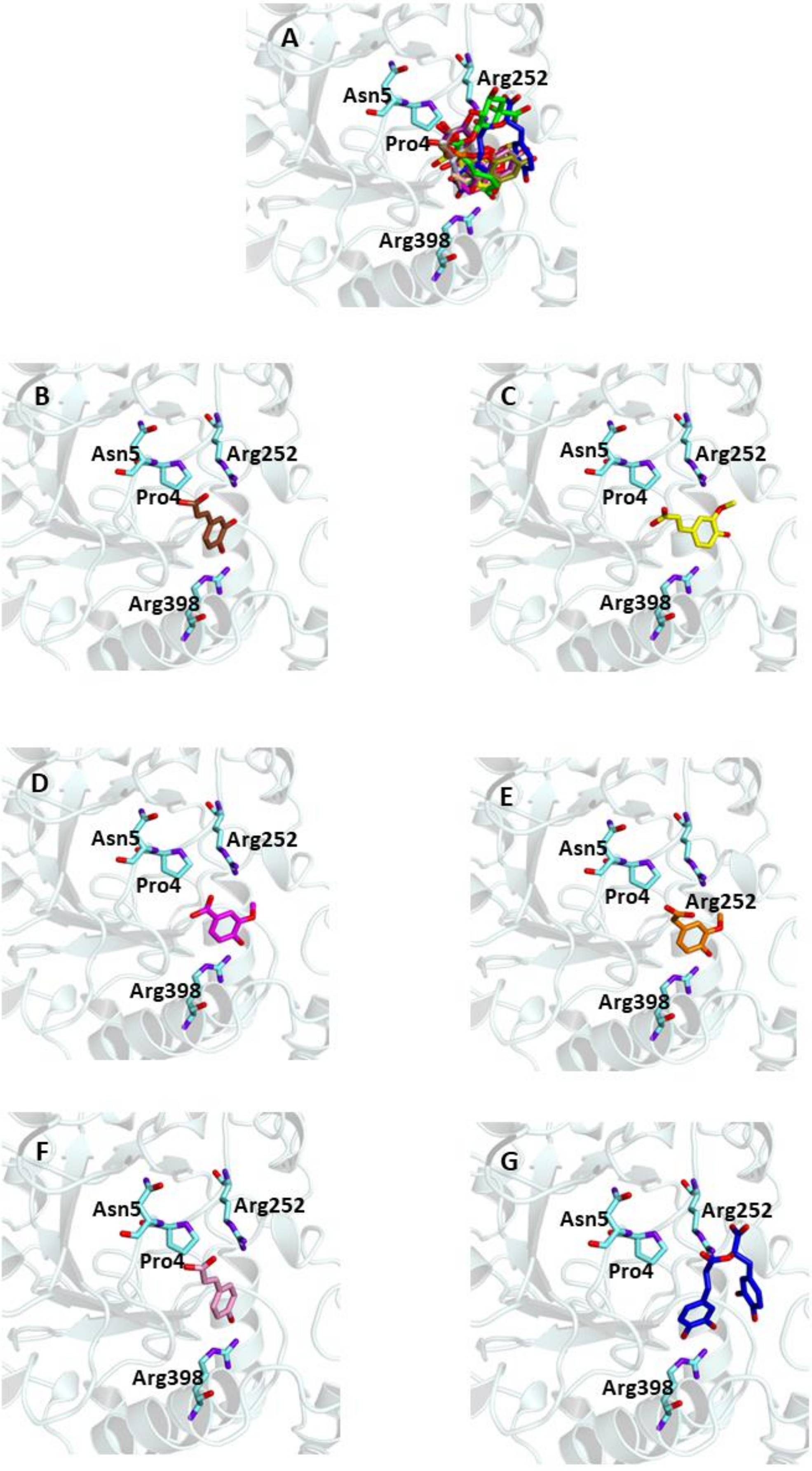
A
Asn5
Arg252
Pro4
Arg398
B
Asn5
Arg252
Pro4
Arg398
C
Asn5
Arg252
Pro4
Arg398
D
Asn5
Arg252
Pro4
Arg398
E
Asn5
Pro4
Arg252
Arg398
F
Asn5
Arg252
Pro4
Arg398
G
Asn5
Arg252
Pro4
Arg398

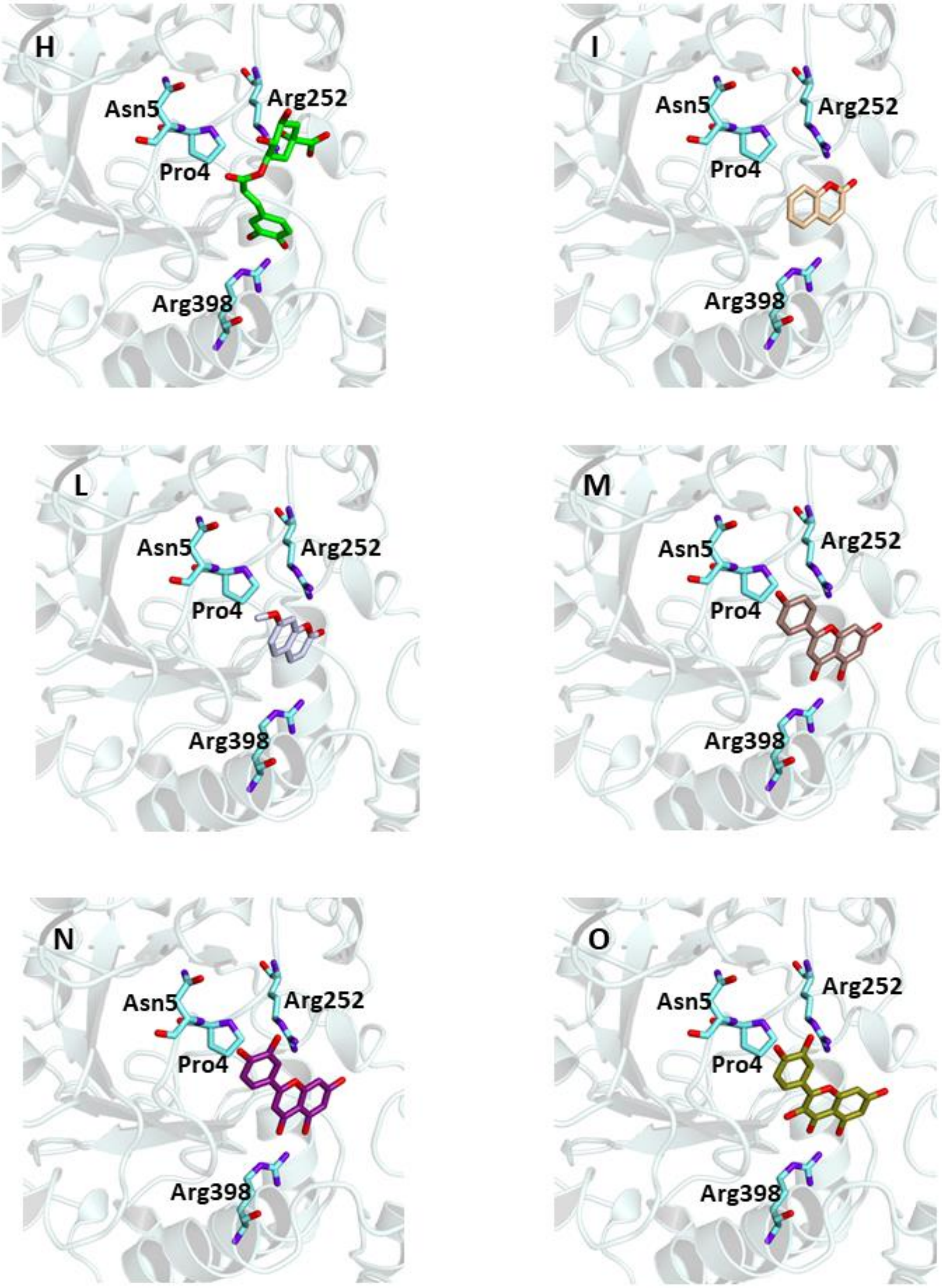

Ligand-binding pocket of the secondary site of α-amylase; ribbons representing protein structural elements are also shown. (**A**) Superimposed binding modes of all the twelve the ligands: caffeic acid (brown), ferulic acid (yellow), vanillic acid (magenta), homovanillic acid (orange), *p*-coumaric acid (pink), rosmarinic acid (blue), chlorogenic acid (green), coumarin (wheat), herniarin (blue-white), apigenin (dark-salmon), luteolin (deep-purple), quercetin (deep-olive); the interaction residues are also indicated in the specific binding mode of (**B**) caffeic acid; (**C**) ferulic acid; (**D**) vanillic acid; (**E**) homovanillic acid; (**F**) *p*-coumaric acid; (**G**) rosmarinic acid; (**H**) chlorogenic acid; (**I**) coumarin; (**L**) herniarin; (**M**) apigenin; (**N**) luteolin; (**O**) quercetin.

Table S4. Complexes binding energy values and protein residues of pancreatic lipase secondary site (1LPB) interacting with the ligands.

LIGAND	Binding Energy Kcal/mol	INTERACTIONS					
		Hydrogen Bonds				Hydrophobic Bonds Residues	Salt Bridges
		Residues	Distance Å		Donar Angle °		
			H-A	D-A			
**Caffeic acid**	-5.9	Leu213	2.94	3.30	104.37	Lys238<br>Asn262	
		Lys239	3.15	3.71	118.29		
		Gln244	2.36	3.21	143.99		
		Asn262	2.96	3.36	105.01		
		His263	2.01	2.97	163.26		
**Ferulic acid**	-5.7	Leu213	3.10	3.44	103.34	Lys238<br>Asn262	
		Gln244	2.65	3.59	158.10		
		Asn262	2.95	3.32	103.53		
		His263	1.93	2.89	163.19		
**Vanilic acid**	-5.1	Gln244	3.22	4.07	144.50	Lys238	
		Asn262	2.65	3.14	111.03		
		Asn262	2.19	3.00	137.95		
		His263	3.05	3.99	158.76		
**Homovanillic acid**	-5.3	Gln244	2.32	3.20	148.29		
		Asn262	2.48	3.13	123.47		
		Asn262	2.68	3.17	110.93		
		His263	2.17	3.13	163.97		
***p*-Coumaric acid**	-5.2	Gln244	2.80	3.30	112.18	Lys238<br>Asn262	
		Asn262	3.02	3.40	104.48		
		His263	2.01	2.96	161.61		
**Rosmarinic acid**	-7.5	Leu213	1.94	2.87	156.12	Phe215	Lys238<br>Lys238
		Phe215	3.11	4.03	156.01		
		Phe258	2.36	2.93	116.74		
		Cys261	2.00	2.96	163.28		
		Asn262	2.43	3.22	136.83		
		Asn262	1.87	2.85	169.89		
**Chlorogenic acid**	-7.4	Asp205	2.51	3.15	123.09	Phe258<br>Asn262	
		Leu213	2.70	3.25	118.25		
		Cys237	2.61	3.18	117.49		
		Lys239	1.99	2.72	128.89		
		Gln244	2.44	2.96	112.23		
		Ala259	2.25	3.16	152.93		
		Cys261	2.52	3.37	144.83		
		Asn262	2.08	3.06	172.70		
**Coumarin**	-6.2	Asn262	2.96	3.40	108.61	Lys238	
		His263	2.18	3.14	165.70		
**Herniarin**	-6.3	Gln244	2.43	2.97	114.55	Phe215	
		Asn262	2.85	3.27	106.95		
		Asn262	3.62	4.10	112.23		
		His263	2.06	3.00	159.39		
**Apigenin**	-7.4	Asp205	2.64	3.53	152.64	Phe258	
		Leu213	2.00	2.89	151.30		
		Asn262	2.78	3.24	109.52		
		His263	2.15	3.11	164.53		
**Luteolin**	-7.7	Leu213	1.93	2.85	153.17	Phe215<br>Phe258	
		Cys237	2.34	2.82	110.20		
		Gln244	2.53	2.92	103.40		
		Ala260	3.50	3.83	103.64		
		Asn262	2.65	3.16	112.53		
		Asn262	3.48	3.91	109.18		
		His263	2.24	3.18	160.81		
**Quercetin**	-7.5	Leu213	2.30	2.71	103.61	Phe258	
		Gln244	2.55	2.93	102.43		
		Asn262	2.70	3.19	111.18		
		His263	2.19	3.15	165.86		

Figure S3

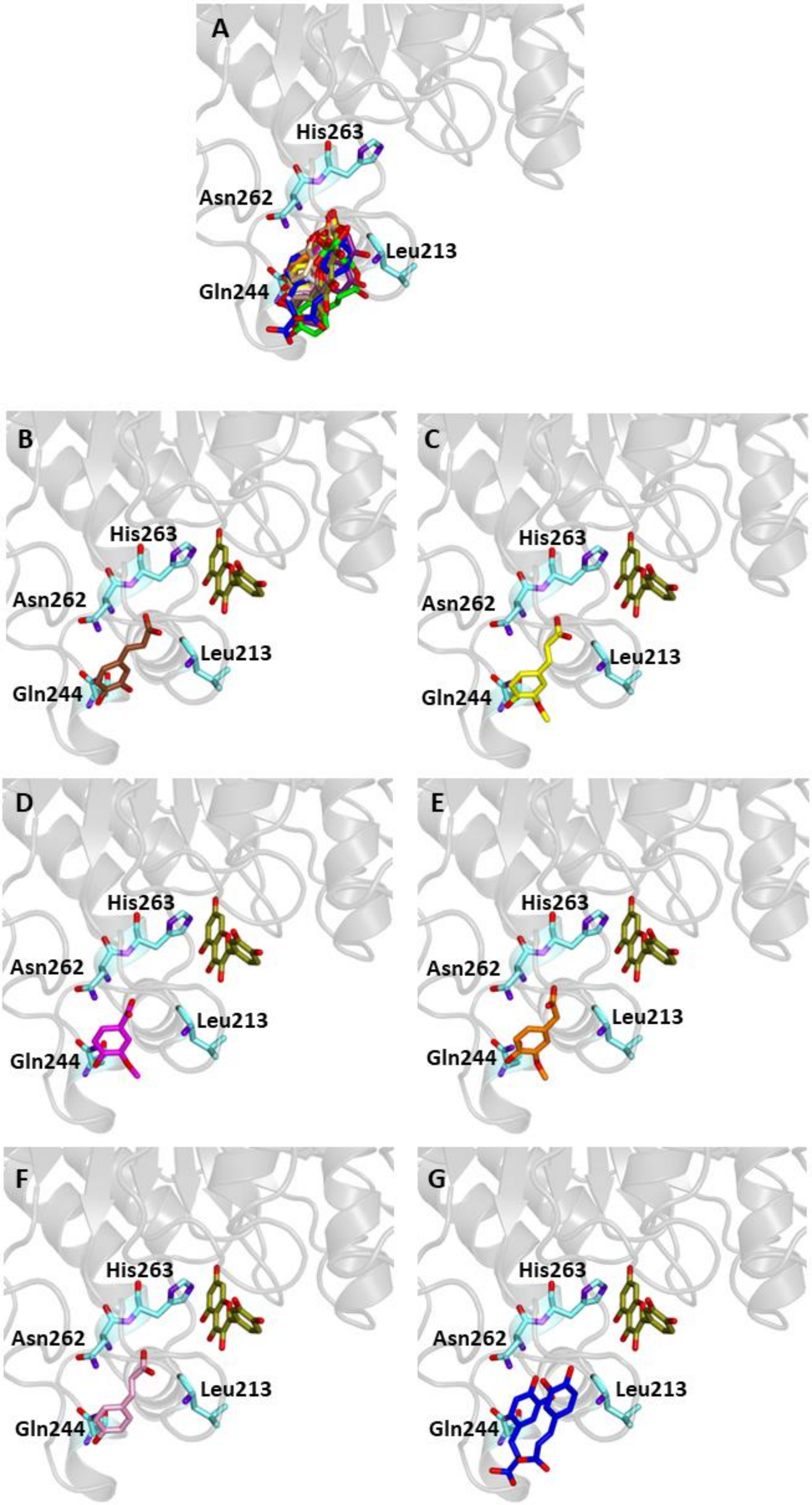

A
His263
Asn262
Leu213
Gln244
B
His263
Asn262
Leu213
Gln244
C
His263
Asn262
Leu213
Gln244
D
His263
Asn262
Leu213
Gln244
E
His263
Asn262
Leu213
Gln244
F
His263
Asn262
Leu213
Gln244
G
His263
Asn262
Leu213
Gln244

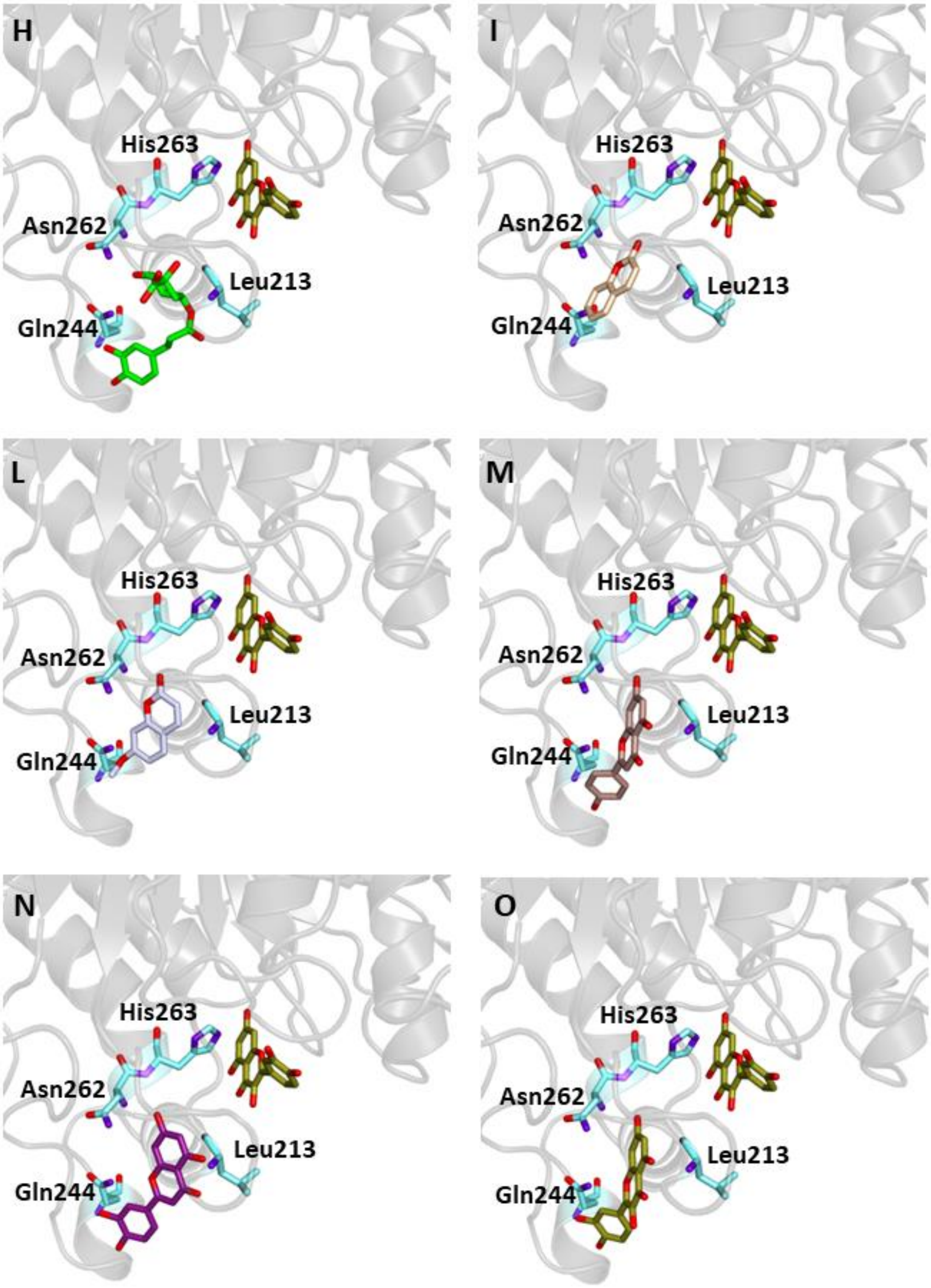

Ligand-binding pocket of the secondary site of pancreattic lipse; ribbons representing protein structural elements are also shown. (**A**) Superimposed binding modes of all the twelve the ligands: caffeic acid (brown), ferulic acid (yellow), vanillic acid (magenta), homovanillic acid (orange), *p*-coumaric acid (pink), rosmarinic acid (blue), chlorogenic acid (green), coumarin (wheat), herniarin (blue-white), apigenin (dark-salmon), luteolin (deep-purple), quercetin (deep-olive); the interaction residues are also indicated in the specific binding mode of (**B**) caffeic acid; (**C**) ferulic acid; (**D**) vanillic acid; (**E**) homovanillic acid; (**F**) *p*-coumaric acid; (**G**) rosmarinic acid; (**H**) chlorogenic acid; (**I**) coumarin; (**L**) herniarin; (**M**) apigenin; (**N**) luteolin; (**O**) quercetin.
